# Evolutionary study of the isoflavonoid pathway based on multiple copies analysis in soybean

**DOI:** 10.1186/1471-2156-15-76

**Published:** 2014-06-24

**Authors:** Shanshan Chu, Jiao Wang, Hao Cheng, Qing Yang, Deyue Yu

**Affiliations:** 1 College of Life Sciences, Nanjing Agricultural University, Weigang 1, Nanjing, 210095, People's Republic of China; 2 National Center for Soybean Improvement, National Key Laboratory of Crop Genetics and Germplasm Enhancement, Nanjing Agricultural University, Weigang 1, Nanjing 210095, People's Republic of China

**Keywords:** Isoflavonoid phytoalexin pathway, Duplication pattern, Evolution divergence, Multiple copies, Soybean

## Abstract

**Background:**

Previous studies suggest that the metabolic pathway structure influences the selection and evolution rates of involved genes. However, most of these studies have exclusively considered a single gene copy encoding each enzyme in the metabolic pathway. Considering multiple-copy encoding enzymes could provide direct evidence of gene evolution and duplication patterns in metabolic pathways. We conducted a detailed analysis of the phylogeny, synteny, evolutionary rate and selection pressure of the genes in the isoflavonoid metabolic pathway of soybeans.

**Results:**

The results revealed that 1) only the phenylalanine ammonia-lyase (*PAL*) gene family most upstream from the pathway preserved all of the ancient and recent segmental duplications and maintained a strongly conserved synteny among these duplicated segments; gene families encoding branch-point enzymes with higher pleiotropy tended to retain more types of duplication; and genes encoding chalcone reductase (CHR) and isoflavone synthase (IFS) specific for legumes retained only recent segmental duplications; 2) downstream genes evolved faster than upstream genes and were subject to positive selection or relaxed selection constraints; 3) gene members encoding enzymes with high pleiotropy at the branching points were more likely to have undergone evolutionary differentiation, which may correspond to their functional divergences.

**Conclusions:**

We reconciled our results with existing controversies and proposed that gene copies at branch points with higher connectivity might be under stronger selective constraints and that the gene copies controlling metabolic flux allocation underwent positive selection. Our analyses demonstrated that the structure and function of a metabolic pathway shapes gene duplication and the evolutionary constraints of constituent enzymes.

## Background

Isoflavonoid phytoalexins are phenolic secondary metabolites. An increasing number of studies have demonstrated that isoflavonoid phytoalexins play an important role in plant defense against pathogens. Isoflavonoid phytoalexins (e.g., medicarpin or glyceollin) are distributed predominantly in leguminous plants and are synthesized through the central phenylpropanoid pathway and legume-specific isoflavonoid branch pathways [1]. To date, plant genetics and biochemical studies have resulted in the isolation and characterization of most of the structural genes involved in phytoalexin production [2,3]. Although the isoflavonoid phytoalexin pathway (to simplify, we refer to it as the isoflavonoid pathway) is one of the most studied secondary metabolic pathways in plants, systematic molecular evolution analyses of isoflavonoid pathway genes in soybeans remain scarce.

In the process of legume divergence, the soybean (2n = 4× = 40) has undergone two rounds of whole genome duplication (WGD) events. The ancient WGD event most likely predated the split between soybean and *Medicago truncatula* (2n = 2× = 16) approximately 50 to 60 million years ago (mya) [4-7], which may have contributed to survival after the Cretaceous-Tertiary extinction event [8]. The recent WGD was found to be an allopolyploidization event that occurred independently in the soybean approximately 5 to 15 mya [8-14]. When whole-genome or chromosome segmental duplication occurs, genes are either lost or retained as repeat genes. Regarding the duplication retention patterns of gene families in metabolic pathways, studies have shown that gene families that encode highly connected enzymes tend to retain more duplicated copies than the gene families related to enzymes with fewer connections [15]. However, no detailed investigation regarding the correlation between gene duplication patterns and the gene’s position or function in metabolic pathways is available.

A primary objective of molecular evolutionary research is to elucidate the driving forces that dominate the variation and mechanisms of molecular evolution. Recently, many studies have focused on how selection acts on the genes involved in metabolic pathways [16,17]. However, controversies related to inconsistencies among research findings exist. For example, several studies have found that genes encoding upstream enzymes were subject to stronger selective constraints and therefore evolved more slowly than genes encoding downstream enzymes [18-22]. However, investigations into the phenylpropanoid pathway in *Arabidopsis thaliana*[23], the gibberellin pathway in the *Oryzeae* tribe [24] and the starch pathway in *Oryza sativa*[25] failed to provide evidence of a correlation between the positions of genes in the pathway and selective constraints or evolutionary rates. In addition, in terms of the branch-point enzymes acting at the center of the metabolic pathways, some theoretical analyses and empirical studies have concluded that adaptive substitutions tend to be concentrated in branch-point genes and therefore tend to be subject to positive selection [26-28]. One opposing observation is that nonsynonymous substitution occurs less frequently in branch-point genes and thus reflects greater selective constraint in these genes [24].

**Figure 1:**
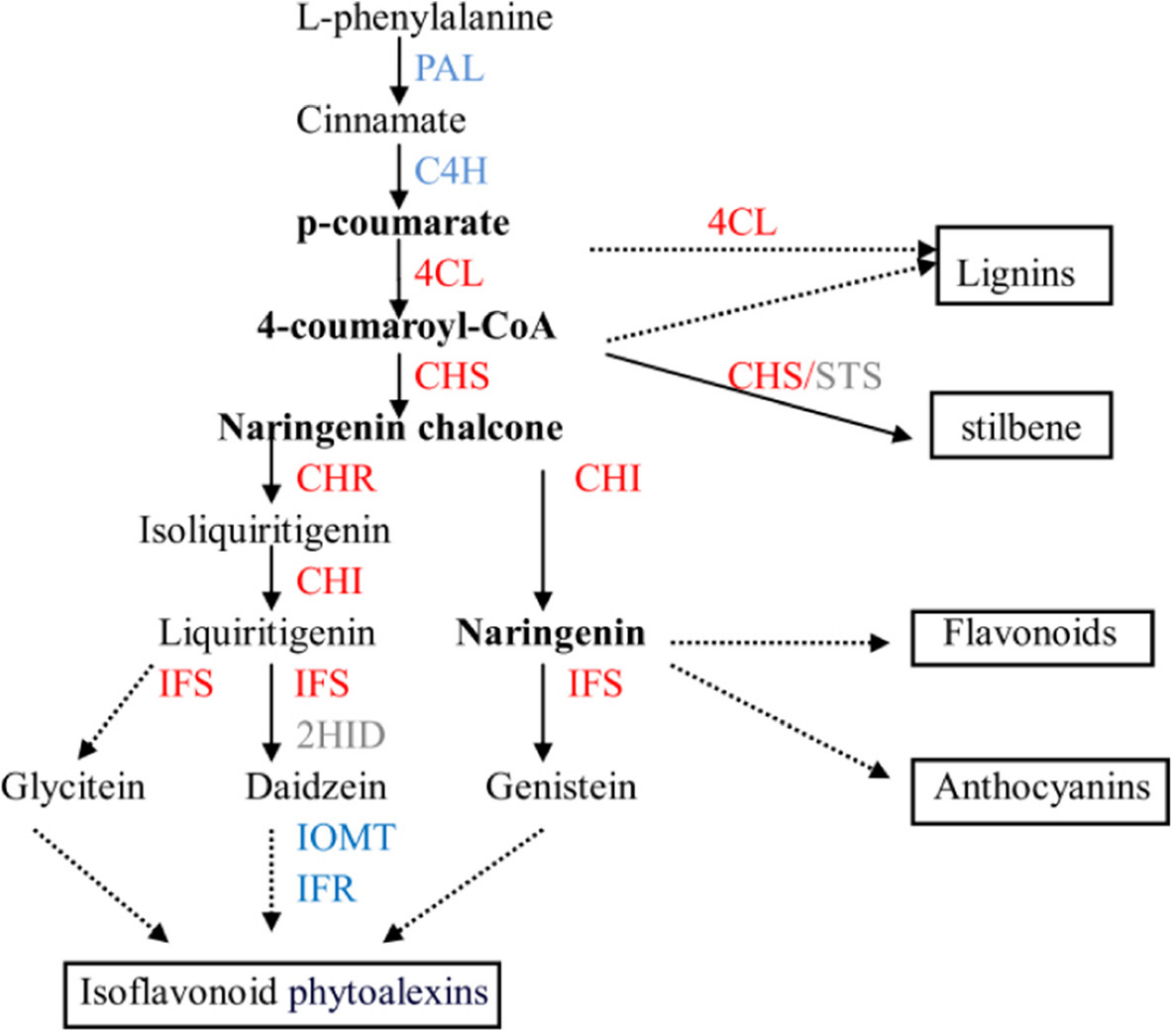
**The isoflavonoid phytoalexin synthesis pathway in soybeans.** Terminal productions of the phenylpropanoid pathway are in brackets. The most upstream and downstream enzymes are indicated in blue. The branch-point enzymes are indicated in red. Metabolic node substances are labeled in bold. Dotted arrows represent multiple or unclear steps.

Most of the abovementioned evolutionary pattern studies have exclusively considered a single gene copy that encoded each enzyme in the metabolic pathway. However, it remains to be determined whether the above conclusions still apply when multiple copies encoding each enzyme are considered. Homologous copies could incur functional differentiation after gene duplication, including pseudogenization [29], sub-functionalization and [30] neo-functionalization [31], which might lead to evolutionary divergence. If only single-copy encoding enzymes are considered in the study of the relationship between evolutionary patterns and the positions of enzymes in the metabolic pathway, bias toward the evolution of metabolic pathway genes might result. Experimental studies have suggested that several gene copies encoding branch-point enzymes located at the central isoflavonoid biosynthetic pathway in soybeans exhibit functional differentiation, such as 4-coumarate:CoA ligase (4CL) and chalcone isomerase (CHI) [32,33]. This phenomenon is expected in soybean due to polyploidy; however, whether there are differential evolution patterns among these functionally differential copies and whether differential evolution patterns are relevant to pleiotropy or to the connectivity of divergent gene copies remain unknown. Above all, considering multiple-copy encoding enzymes in the study of differential evolutionary patterns of genes in metabolic pathways could provide clearer evidence of the way selection power acts on genes in metabolic pathways than previous studies have offered.The isoflavonoid phytoalexin pathway in soybeans provides an excellent system for investigating the influence of metabolic pathway structure on the duplication and evolutionary patterns of enzymes. First, multiple copies of gene-encoding enzymes in the isoflavonoid biosynthetic pathway were retained as the soybean experienced two rounds of WGD events. Second, the pathway contains nine major enzymes acting at different positions and four metabolic nodes of the isoflavonoid biosynthesis pathway. Therefore, the network topology is suitable to investigations of the effect that the positions and functions of enzymes in the pathway on gene duplication and evolutionary patterns (Figure 1). Metabolic node substances are defined as the substances that participate in two or branching pathways. Branch-point enzymes are the enzymes that catalyze these reactions and primarily include4-coumarate:CoA ligase (4CL), chalcone synthase (CHS), chalcone reductase (CHR), CHI and isoflavone synthase (IFS). In general, 4CL participates in most of the pathways involved in the biosynthesis of lignin, flavone, flavonol, anthocyanin and isoflavonoid. CHS and CHI also participate in these pathways, with the exception of the lignin biosynthesis pathway. In contrast, CHR and IFS only control isoflavonoid biosynthesis. Therefore, we grouped 4CL, CHI and CHS into enzymes with greater pleiotropy and CHR and IFS into enzymes with less pleiotropy in the branching pathway. To avoid ambiguous boundaries in the branching pathway, the most upstream enzymes, phenylalanine ammonia-lyase (PAL) and cinnamate 4-hydroxylase (C4H), are classified as the upstream enzymes, whereas the most downstream enzymes, isoflavone O-methyltransferase (IOMT) and isoflavone reductase (IFR), are downstream enzymes.

Considering these enzymes together, we identified the isoflavonoid biosynthesis pathway genes in the soybean genome to investigate duplication and evolutionary patterns by analyzing the phylogenies, synteny, evolutionary rate and selection pressure of the genes in each gene family. We sought to examine the following: 1) whether different duplicate retention patterns exist in different gene families and how pathway position, node connectivity and pleiotropy affect the duplicate retention patterns; 2) whether positive selection signatures and evolutionary rate heterogeneity exist in genes that encode the enzymes involved in the isoflavonoid biosynthesis pathway and how they are distributed in this pathway; and 3) whether differential evolutionary patterns exist among multiple gene copies that encode each enzyme involved in the isoflavonoid biosynthesis pathway in soybeans.

## Results

### Gene duplication patterns in isoflavonoid pathway gene families

The isoflavonoid pathway gene sequences were used to search against seven plant genome databases. *PAL*, *C4H*, *4CL*, *CHS* and *CHI* were detected in all flowering plants surveyed, whereas *CHR*, *IFS*, *IOMT* and *IFR* were specific to legumes (Table 1). The copy number for each gene varied among plants. A maximum of a five-fold size difference existed in *CHS*, and 4 and 21 homologs were found in *A. thaliana* and *M. truncatula*, respectively. Diverse copy numbers were also detected among genes. There were 7 *PAL*, 3 *C4H*, 6 *4CL*, 12 *CHS* and 6 *CHI* homologous genes, on average, in the plants we surveyed. In addition, the copy numbers of *CHR*, *IFS*, *IOMT* and *IFR* were 4, 3, 10 and 6, respectively. Clearly, the number of *CHR* and *IFS* genes was significantly less than the number of *IOMT* and *IFR* genes.

**Table 1:** The number of genes of each gene family in the isoflavonoid pathway in various plants and the number of ancestral genes in the most recent common ancestor (MRCA) of different lineages

	***Glycine max***	***Phaseolus vulgaris***	***Medicagotruncatula***	***Cicerarietinum***	***Arabidopsis thaliana***	***Vitisvinifera***	***Oryza sativa***	**Average**	**Legumes**^**a**^	**Dicots**^**b**^	**Dicot/monocot**^**c**^
*PAL*	8	6	6	5	4	11	9	7	4	1	1
*C4H*	4	3	2	3	1	2	4	3	3	2	2
*4CL*	9	5	4	6	6	4	7	6	5	4	3
*CHS*	13	11	21	4	4	14	15	12	4	3	2
*CHI*	8	7	9	5	5	3	4	6	5	3	3
*CHR*	2	2	4	6	0	0	0	4	1	0	0
*IFS*	2	3	3	2	0	0	0	3	1	0	0
*IOMT*	17	6	12	4	0	0	0	10	3	0	0
*IFR*	8	7	4	6	0	0	0	6	5	0	0

^a^Ancestral genes in the MRCA of legumes.

^b^Ancestral genes in the MRCA of dicots.

^c^Ancestral genes in the MRCA of dicots and monocots.

**Figure 2:**
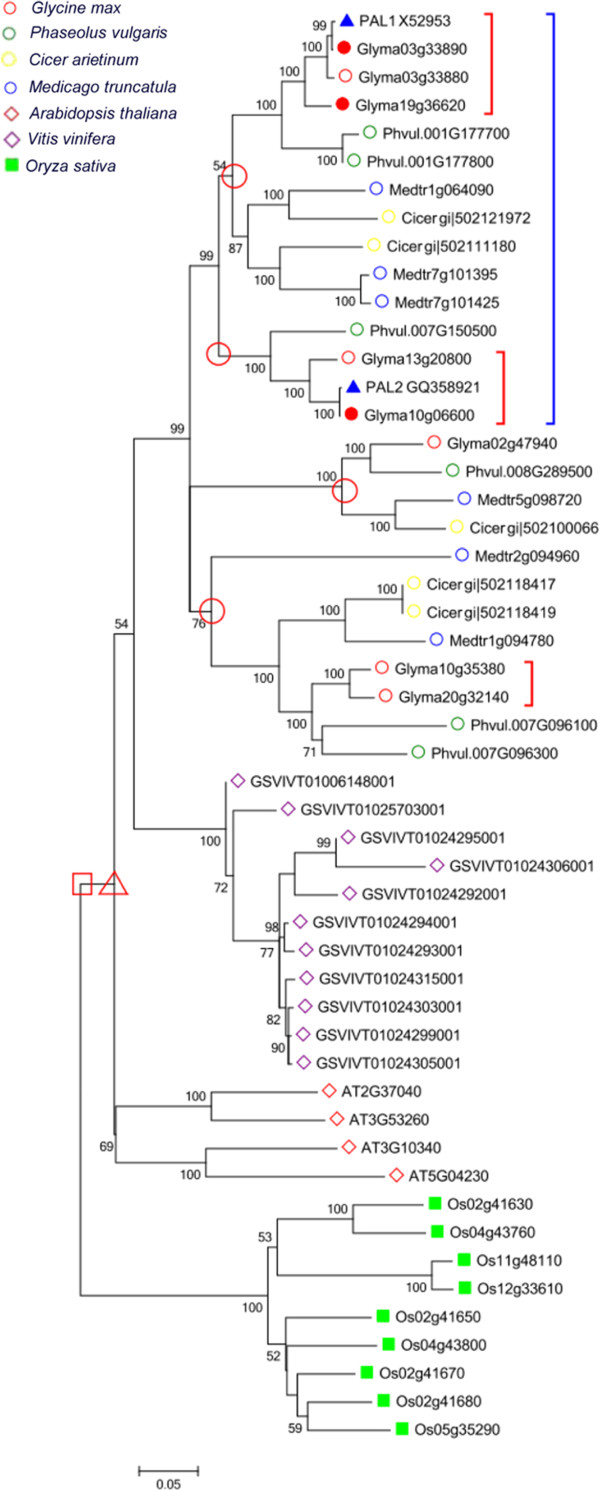
**Phylogenic relationship of the *PAL *gene family members from 7 species.** Each species was labeled with different shapes as presented in the figure. Genes reported from the NCBI are highlighted with a blue triangle. The genes sequenced in our study are highlighted with a red dot. The red circles at the nodes represent ancestral genes in the MRCA of legumes. The red triangles and rectangles represent ancestral genes in the MRCA of dicots and those of dicots and monocots, respectively. The nodes with bootstrapping lower than 50% are not shown. Each red square bracket represents one recent segmental duplication; each blue square bracket represents one old segmental duplication.

Phylogenetic analyses allow us to identify the evolutionary history of the isoflavonoid pathway genes. Genes from legumeswere clustered in one clade. Homologs from *A. thaliana* and *Vitis vinifera* were located outside of legumes, and homologs from rice gathered in the outermost regions. Therefore, the gene trees containing different flowering plants were congruent with the species phylogeny. To better understand how these genes evolved in plants, we estimated the number of isoflavonoid pathway genes in the most recent common ancestor (MRCA) of dicots and monocots, that of dicot and that of legumes, respectively (Table 1, Figure 2 and see Additional file 1: Figure S1). Reconciliation of the gene trees for *PAL*, *C4H*, *4CL*, *CHS* and *CHI* revealed 1, 2, 3, 2 and 3 ancestral genes, respectively, in the MRCA of dicots and monocots. When the numbers of ancestral genes were compared with the gene numbers in each plant, it appeared that the expansion was uneven among gene families. The average size of *C4H*, *4CL* and *CHI* increased approximately 1.4- to 2-fold after the dicot/monocot split ~145 mya. In contrast, dramatic expansions occurred in *PAL* and *CHS*, the size of which increased 7- and 6-fold, respectively, since the divergence of dicots and monocots. The numbers of *PAL*, *C4H*, *4CL*, *CHS* and *CHI* genes in the MRCA of dicots are similar to those in the MRCA of dicots and monocots, indicating that no major expansion occurred before the divergence of dicots. When the numbers of isoflavonoid pathway genes in the MRCA of legumes were compared with those in each legume, uneven expansions were also detected among gene families. *CHS*, *CHR* and *IOMT* have tripled in size since the split of legumes ~54 mya [34]. In contrast, the sizes of other families have been relatively stable since the legume split.

**Figure 3:**
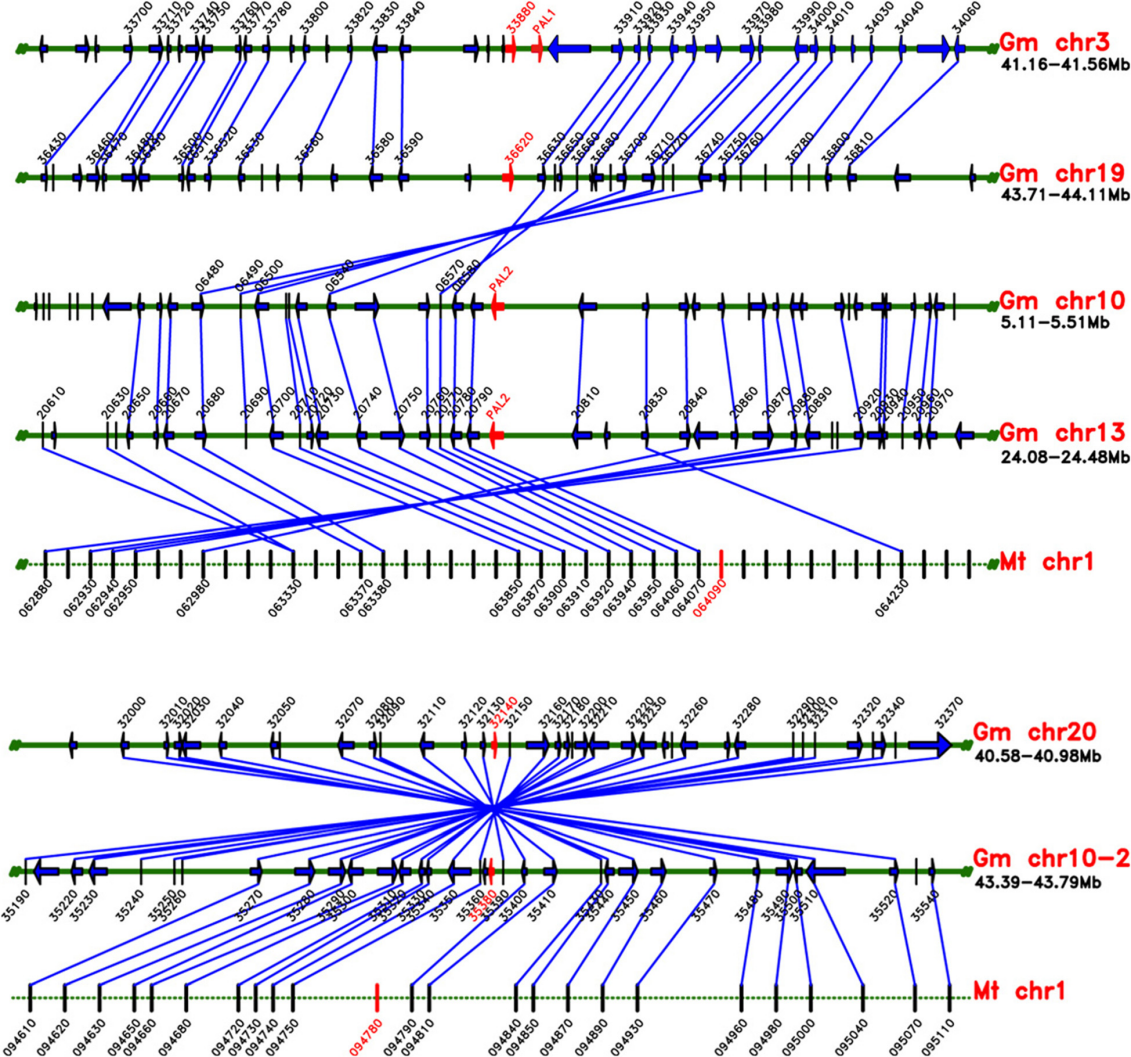
**Synteny analyses for the *PAL *gene family.** Green solid and dotted lines represent soybean and *M. truncatula* chromosomes, respectively. Target genes are indicated by red arrows.

To more precisely investigate the duplication patterns of isoflavonoid pathway genes, the physical locations of all homologs of each family were positioned and categorized as segmental or tandem duplications. On one hand, we conducted synteny analysis and dated segmental duplication events (Figure 2, Table 2 and see Additional file 2: Figure S2). Segmental duplications following the first round of WGD (50 to 60 mya) and the second round of WGD (5 to 15 mya) were classified into old and recent segmental duplications, respectively. On the other hand, we classified tandem duplications that occurred before Leguminosae differentiation (duplications shared by soybeans and *M. truncatula*) as old tandem duplications and those that were specific to soybeans as recent tandem duplications. We summarized the recent and old duplications among each gene family (Table 3). Old segmental duplications were exclusively detected in the most upstream gene family, *PAL*. Recent duplications occurred at each of the old segmental duplicated genes. Finally, the *PAL* gene family retained both old and recent segmental duplications. Meanwhile, a perfect synteny relationship was detected among these duplicated genes (Figure 3). Recent segmental duplication occurred approximately four times in the *CHS* gene family and three times each in *PAL*, *4CL* and *CHI*; in contrast, the *CHR* and *IFS* gene families only retained copies from one to two recent segmental duplications. Old tandem duplication occurred once and twice in the *CHI* and *IFR* gene families, respectively, and all of the old duplications were retained in both soybeans and *M. truncatula* during the Leguminosae evolution process. One old tandem duplication was detected in the *4CL* gene family, but one of the tandem duplicated genes was lost in *M. truncatula*. Recent tandem duplication occurred five times in both the *CHS* and *IOMT* gene families; thus, those families contained the greatest number of gene copies. The recent tandem duplication of the *IOMT* gene family occurred before the second segmental duplication event. Unlike the *IOMT* gene families, however, the recent tandem duplications in *CHS, PAL, 4CL* and *IFR* occurred after the recent segmental duplication.

**Table 2:** Date calculations for segmental duplication events in soybeans

**Segment pairs**	**Number of anchors**	**K**_**S **_**(mean ± s.d.)**	**Estimated time (mya)**
Segments containing *PAL* paralogs			
Chr 3 & Chr 19	10	0.17 ± 0.04	14
Chr 10 & Chr 13	10	0.14 ± 0.03	11
Chr 3 & Chr 10	5	0.60 ± 0.15	49
Chr 3 & Chr 13	5	0.62 ± 0.25	51
Chr 19 & Chr 10	4	0.58 ± 0.05	48
Chr 19 & Chr 13	4	0.66 ± 0.22	54
Chr 10–2 & Chr 20	10	0.12 ± 0.02	10
Segments containing *C4H* paralogs			
Chr 2 & Chr 14	10	0.14 ± 0.02	11
Chr 10 & Chr 20	10	0.15 ± 0.03	12
Segments containing *4CL* paralogs			
Chr 17 & Chr 13	10	0.16 ± 0.08	13
Chr 13–2 & Chr 15	10	0.12 ± 0.04	10
Chr 1 & Chr 11	4	0.10 ± 0.04	8
Segments containing *CHS* paralogs			
Chr 8 & Chr 5	10	0.14 ± 0.05	11
Chr 1 & Chr 2	8	0.17 ± 0.07	14
Chr 1–2 &Chr 11	10	0.10 ± 0.01	8
Segments containing *CHR* paralogs			
Chr 14 &Chr 2	10	0.12 ± 0.04	10
Segments containing *CHI* paralogs			
Chr 10 & Chr 20	10	0.18 ± 0.13	15
Chr 4 & Chr 6	10	0.17 ± 0.05	14
Chr 13 & Chr 15	7	0.21 ± 0.10	17
Segments containing *IFS* paralogs			
Chr 7 & Chr13	10	0.18 ± 0.11	15
Segments containing *IOMT* paralogs			
Chr 18 & Chr 8	10	0.22 ± 0.11	18
Chr 10 & Chr 20	10	0.14 ± 0.03	11
Segments containing *IFR* paralogs			
Chr 1 & Chr11	10	0.18 ± 0.13	15

*Abbreviation*: mya, million years ago.

**Table 3:** Gene duplication patterns of gene families in the isoflavonoid biosynthesis pathway

**Duplication types**		**PAL**	**C4H**	**4CL**	**CHS**	**CHR**	**CHI**	**IFS**	**IOMT**	**IFR**
Segment duplication^a^	Old	1								
	Recent	3(6)^c^	2(4)	3(6)	4(7)	2(3)	3(6)	1(2)	2(4)	2(3)
Tandem duplication^b^	Old			1			2			1
Total number of duplications^d^	Recent	1	4	2	5	4	8	2	5	2
		8		9	12				14	9

^a^The total number of segment duplications.

^b^The total number of tandem duplications.

^c^The number in the parentheses is the number of loci retainedafter recent segment duplication; Tandemly duplicated genes were considered one locus.

^d^The total number of gene copies originating from segmental and tandem duplications.

### Comparing π, d_N_ and d_S_ in upstream and downstream genes

Partial coding sequences of the twenty-one gene members from the eight gene families involved in the isoflavonoid biosynthesis pathway were isolated from 33 sampled Chinese soybean accessions; additional sequence information for two gene copies of *IFS* was also used in this study [35]. The π, d_N_ and d_S_ values in the upstream and downstream genes are listed in Table 4. Upon comparison of polymorphism and evolutionary rates among the different gene copies encoding each upstream and downstream enzyme, similar levels of these parameters were observed among these multiple copies. Next, we compared the evolutionary patterns of the upstream and downstream genes. The π values ranged from 0.00 (cinnamate 4-hydroxylase [*C4H*]) to 0.16 (*PAL1*) in the upstream gene copies. In contrast, the values ranged from 0.20 (*IFR*) to 0.44 (*IOMT*) in the downstream gene copies. The average π value of all of the downstream gene copies (0.28) was four-fold higher than that of all upstream gene copies (0.07) in the pathway (Table 4). This finding suggests that the rate of nucleotide polymorphism in the downstream genes was significantly increased compared with the upstream genes (P < 0.05). The d_N_ value also varied greatly between the upstream and downstream genes; values ranged from 0.18 (*IOMT*) to 0.37 (*IOMT)* in the downstream genes compared with 0 for three gene members to 0.02 (for *PAL2*) in the the upstream genes. The average d_N_ value of all of the downstream gene copies (0.29) was approximately 30-fold higher than all upstream gene copies (0.01) in the pathway. However, the average d_S_ values were similar for the upstream (0.28) and downstream genes (0.26).

**Table 4:** Differential evolutionary pattern between upstream and downstream genes

**Pathway position**	**Enzyme**	**Gene locus**	**π(%)**	**Average π(%)**	**P-value**^**a**^	**d**_**N**_**(%)**	**Average d**_**N**_**(%)**	**P-value**^**b**^	**d**_**S**_**(%)**	**Average d**_**S**_**(%)**	**P-value**^**c**^	**d**_**N**_**/d**_**S**_	**LRT**
Upstream	PAL	Glyma19g36620(*PAL1*)	0.16	0.07	0.04*	0.01	0.01	0.01**	0.63	0.28	0.46	0.02	0.00
		Glyma03g33890(*PAL1*)	0.03			0.02			0.08			0.28	0.00
		Glyma10g06600(*PAL2*)	0.07			0.00			0.30			0.00	0.00
	C4H	Glyma14g38580	0.09			0.00			0.41			0.00	0.00
		Glyma02g40290	0.00			0.00			0.00			0/0	0.00
Downstream	IOMT	Glyma13g24210	0.22	0.28		0.18	0.29		0.35	0.26		0.51	0.00
		Glyma18g50290	0.44			0.37			0.68			0.54	0.00
	IFR	Glyma01g37840	0.20			0.26			0.00			0.26e-2/0	1.13
		Glyma04g01380	0.27			0.35			0.00			0.35e-2/0	25.15**

^a^T-test of the π values between upstream and downstream genes.

^b^T-test of thed_N_ values between upstream and downstream genes.

^c^T-test of thed_S_ values between upstream and downstream genes.

LRT statistics are twice the log-likelihood differences between M7 and M8.

*0.01 < P < 0.05; **0.001 < P < 0.01.

### Divergent evolutionary patterns of genes encoding branch-point enzymes

Regarding branch-point enzymes that are centrally located in the metabolic pathway, we observed divergent evolutionary patterns among different gene copies encoding each high pleiotropic branch-point enzyme (*4CL*, *CHS*, *CHI*) regardless of the level of polymorphism or the evolutionary rate (Table 5). For example, the π values of multiple copies encoding CHI varied greatly from 0.00 (*CHI1B2* and *CHI4*) to 0.79 (*CHI3*), and the d_N_/d_S_ ratio of multiple copies encoding CHS ranged widely from 0.00 (*CHS8*) to 1.49 (*CHS2*). In contrast, no significant differences in evolutionary pattern between different copies encoding the less-pleiotropic branch-point enzymes (CHR, IFS) were observed.

**Table 5:** Evolutionary pattern of genes encoding for branch-point enzymes

**Pleiotropy**	**Enzyme**	**Gene locus**	**π(%)**	**d**_**N**_**(%)**	**d**_**S**_**(%)**	**d**_**N**_**/d**_**S**_	**LRT**
Higher	4CL	Glyma17g07190(*4CL1*)	0.18	0.14	0.30	0.48	0.00
		**Glyma13g44950( *4CL2 *)**	0.40	0.39	0.40	0.96	28.98**
		**Glyma11g01240( *4CL3 *)**	0.09	0.12	0.00	0.12e-2/0	66.60**
	CHS	**Glyma05g28610( *CHS2 *)**	0.17	0.18	0.12	1.49	112.46**
		Glyma01g43880(*CHS7*)	0.03	0.04	0.00	0.04e-2/0	0.00
		Glyma11g01350(*CHS8*)	0.07	0.00	0.29	0.00	0.00
	CHI	Glyma10g43850(*CHI1B2*)	0.00	0.00	0.00	0/0	0.00
		**Glyma20g38580( *CHI2 *)**	0.40	0.23	0.97	0.24	60.41**
		Glyma06g14820(*CHI4*)	0.00	0.00	0.00	0/0	0.00
		Glyma13g33730(*CHI3*)	0.79	0.58	1.49	0.39	0.40
Lower	CHR	Glyma02g47750	0.27	0.00	1.29	0.00	0.00
		Glyma14g00870	0.11	0.07	0.25	0.30	0.00
	IFS	Glyma07g32330(*IFS1*)	0.22	0.12	0.56	0.21	0.00
		Glyma13g24200(*IFS2*)	0.17	0.12	0.32	0.38	0.00

LRT statistics are twice the log-likelihood differences between M7 and M8.

Significant positive sites are labeled in bold.

*0.01 < P < 0.05; **0.001 < P < 0.01.

### Detection of positive selection

A significant rate of heterogeneity among the isoflavonoid biosynthesis pathway genes, particularly for d_N_/d_S_ ratios that span more than one order of magnitude, might result either from the intense purifying selection on slowly evolving genes (e.g., *PAL*) or from the frequent episodes of positive selection or relaxed purifying selection on fast-evolving genes (e.g., *4CL, CHS*). Instead of overall d_N_/d_S_ ratios that detect accumulated mutations across the entire gene region, maximum likelihood analyses of ω conducted by PAML [36] can effectively reveal positive selection acting among specified sites. This analysis revealed that significantly positive sites existed in many of the genes that encode enzymes in the isoflavonoid biosynthesis pathway, such as *4CL2, 4CL3, CHS2, CHI2* and *IFR2* (Tables 4 and 5). The existence of these sites indicates that positive selection tended to occur in branch-point enzymes, especially those with higher pleiotropy.

## Discussion

### Duplication pattern of gene families in the isoflavonoid pathway

Our study investigated the retention patterns of duplicated genes involved in the isoflavonoid biosynthesis pathway following two WGD events in soybeans and attempted to determine whether any correlation exists between a gene family’s duplication pattern and its position or function in the pathway. In our study, only the *PAL* gene family, which was the most upstream of the entire pathway, preserved all of the ancient and recent segmental duplications and maintained a strongly conserved synteny among these duplicated segments. PAL is the entry point enzyme of the phenylpropanoid pathway and directly controls the production of multiple secondary metabolites downstream. PAL plays important roles in plant development and variable environment responses. Regarding the perfect synteny maintained among *PAL* gene regions in soybean, we suggest that the chromosome regions flanking the *PAL* genes were very stable to ensure the gene’s functional stability.

Although the recent segmental duplication was preserved in all gene families in the soybean isoflavonoid synthetic pathway, the number of duplications was asymmetrically distributed in the branching pathway. Gene families encoding enzymes with higher pleiotropy tend to retain more recent segmental duplications (3 to 4) than those with lower pleiotropy (1 to 2). In addition to recent segmental duplications, gene families with higher pleiotropy reserved more gene copies from other types of duplication, such as the old tandem duplication in *4CL* and *CHI* and therecent tandem duplication in *4CL* and *CHS*. In comparison, branch-point gene families with lower pleiotropy, such as *CHR* and *IFS*, only retained 4 and 2 copies, respectively, from recent segmental duplication. We hypothesized that enzymes with higher pleiotropy catalyze metabolic node substances that are responsible for a greater range of downstream production and thus required more copies to reinforce their function. However, both *CHR* and *IFS*, which are legume-specific genes, play a pivotal role in isoflavonoid biosynthesis. An increased number of copies and copy duplication types were detected in one of the downstream enzymes, *IOMT* (14 copies). A previous study revealed that recombinant *M. truncatula IOMT*s had the ability to catalyze differential conformational substrates [37]; thus, we inferred that more copies were needed to maintain distinct catalytic properties.

### Relationship of substitution rate and selection to pathway structure

Evolutionary patterns between upstream and downstream genes were significantly distinct; the average π value and nonsynonymous substitution rate were significantly increased in downstream genes, whereas the synonymous substitution rate was similar between the upstream and downstream genes. This result indicated that downstream genes evolved faster than upstream genes, and this phenomenon is potentially not attributable to mutation rates given the similar synonymous substitution rates. A possible interpretation is that downstream enzymes are subject to positive selection or relaxed selection. Consistently, positive selection was detected in the gene-encoding downstream IFR enzyme. Increased nonsynonymous substitution rates in downstream genes compared with upstream genes were also observed in anthocyanin pathway genes in *Ipomoea*. This finding was attributed to relaxed constraints on the downstream genes rather than positive selection, as the investigators failed to detect positive selection in this pathway [20,21]. In comparison, these two selection pressures were thought to influence the nucleotide patterns in the carotenoid and terpenoid pathway [18,22]. Two explanations potentially account for the phenomenon that upstream enzymes evolved more slowly than downstream enzymes and were subject to strong purifying selection [22]. First, upstream enzymes evolve to exert more control over metabolic fluxes than downstream enzymes [17,38]. Second, upstream enzymes influence a larger number of pathway end products than downstream enzymes [16,18,19]. Nevertheless, stronger selective constraints on upstream genes cannot be applied to all metabolic pathways. For example, no correlation was detected between pathway position and various estimates of nucleotide divergence of the genes in the gibberellin and starch pathways in rice [24,25] or in the phenylpropanoid metabolism pathway in *A.thaliana*[23]. This absence of correlation was potentially observed because the researchers considered branch-point enzymes and did not compare the most upstream enzymes with the most downstream enzymes. In our study, the different gene copies encoding branch-point enzymes tended to exhibit differential evolution rates and selective constraints. For this reason, considering a single copy when branch-point enzymes are coded by multiple copies might affect the comparison.

### Evolutionary pattern of branch-point enzymes

According to our study, the gene members encoding the enzymes (such as 4CL, CHS, and CHI) with high pleiotropy at the branching points tend to retain more gene copies. These duplicated copies are more likely to have evolutionary differences, corresponding to their functional divergences. Divergent members of the *4CL* gene family have been identified in soybeans and exhibited pronounced differences in the ability of the isoenzymes to catalyze different substrates [32]. For example, 4CL catalyzes the reaction of p-coumarate to produce the final products flavone, flavonol, anthocyanin and isoflavonoid. In addition, the recombinant 4CL1 isoform could utilize several other substrates (such as ferulate and sinapate) to channel flux to the lignin biosynthetic pathway. Given that it accepted the broadest range of substrates, *4CL1* was shown to be under purifying selection. In contrast, positive selection was detected in *4CL2* and *4CL3*, which acted on fewer substrates with great efficiency.

Previous efforts to compare global gene expression in two soybean cultivars with different seed isoflavonoid contents have demonstrated that the *CHS7* and *CHS8* genes play significant roles in isoflavonoid synthesis [39]. The gene loci corresponding to *CHS7* and *CHS8* were subject to strong purifying selection. In comparison, *CHS2* was under positive selection, as demonstrated by a d_N_/d_S_ value greater than 1 and the existence of positive sites in PAML analysis.

Functional divergence has also been experimentally proven to exist in the *CHI* enzyme, as indicated by differences in the level of expression and kinetics among the various types of soybeans [33]. For instance, *CHI1B2* members belonging to Type II CHI use a variety of chalcone substrates and are coordinately regulated with an isoflavonoid-specific gene, whereas *CHI2* members belonging to Type I CHI exclusively use naringenin chalcone as the substrate and are coordinately regulated with other flavonoid-specific genes. In our study, *CHI1B2* underwent purifying selection, whereas *CHI2* was under positive selection. This observation was consistent with the conclusion that gene copies with an ability to utilize a larger range of substrates (*4CL1,CHI1B2*) underwent purifying selection, whereas those using a narrower range of substrates (*4CL2, 4CL3 and CHI2*) underwent positive selection. A reasonable explanation could be that the gene copies at branch points with wide-ranging catalytic activities and high connectivity are under stronger selective constraint [15,24]. In addition, greater pleiotropy, which is often believed to experience stronger selective constraint [18], potentially plays a role in the higher connectivity in branch-point enzymes in the isoflavonoid pathway. The gene copies (*4CL2*, *4CL3* and *CHI2*) that catalyze a narrower range of substrates, mainly leading to the production of 4-coumaroyl-CoA and naringenin chalcone in the metabolic branch, potentially play more important roles in flux allocation [22]. Our study results reinforced previous findings that branch-point enzymes were the targets of positive selection in the central metabolism of *Drosophila*[27], the starch pathway in maize [28] and the asparagine N-glycosylation metabolic pathway among human populations [40]. These results provided further evidence that positive selection events were asymmetrically distributed in multiple-copy genes encoding branch-point enzymes and were concentrated on gene copies with more powerful control over metabolic flux allocation. We hypothesized that these positive selection events contribute to higher isoflavonoid content in soybean or legumes. This hypothesis requires further investigations comparing a large number of legumes or non-legume species.

### The evolutionary characteristics of gene copies closely related to isoflavonoid biosynthesis

We originally hypothesized that legume-specific genes that directly contribute to isoflavonoid content should be subject to positive selection during legume evolution. However, we failed to detect positive sites in the genes encoding the CHR and IFS enzymes, which play a pivotal role in isoflavonoid biosynthesis and have been reported to be specific to legumes [41,42]. A possible explanation could be that we only conducted positive selection tests in *Glycine* without considering other taxonomic groups. To adapt to new environments, legume-specific genes might be subject to positive selection during the early evolution stage. Thereafter, purifying selection might affect these genes and create functional stability with the completion of function evolution [43-46].

Surprisingly, we also observed that the gene copies that were more heavily involved in isoflavonoid synthesis (*CHI1B2*, *CHS7* and *CHS8*) and that the legume-specific genes (*CHR* and *IFS*) directly controlling the production of isoflavonoids were all subject to purifying selection. We propose that these enzymes in the isoflavonoid pathway experienced convergent evolution and were subject to similar selection. This hypothesis requires substantial functional evidence in plants for confirmation.

## Conclusions

Although numerous evolutionary studies on pathway genes have been performed, evidence on the duplication pattern of multiple-copy gene families is lacking. The results of this study provide evidence that there exists correlation between a gene family’s duplication as well as evolutionary patterns and its position or function in the pathway. It is noteworthy that gene copies encoding branch-point enzymes with high pleiotropy tend to possess evolutionary divergence and undergo many duplication events. This result underscores the need for multicopy-based approaches in studies of the molecular evolution of metabolic pathways. Interestingly, the evolutionary differentiation of gene copies located at branch points potentially corresponds to their functional divergences (e.g., the gene copies closely related to isoflavonoid synthesis were all subject to purifying selection). More intensive molecular evolution studies on multiple gene copies involved in this pathway would offer profound insight for engineering isoflavonoid composition in soybean.

## Methods

### Sequence collection

Seven plant genomes were used to identify the isoflavonoid synthesis pathway genes. Their sequences and corresponding annotations were downloaded from online databases (see Additional file 3: Table S1). The sequences used to generate the BLAST results were the reported coding DNA sequences (CDS) of putative soybean isoflavonoid synthesis genes; the exception was isoflavone O-methyltransferase (*IOMT*) gene, which has been identified only in *M. truncatula* (see Additional file 3: Table S2). Both BLASTn and BLASTp were conducted for non-legumes with cutoff E values of 1e-20 and 1e-100, respectively; BLASTn was conducted for legumes, with a cutoff E value of 1e-20.

### Phylogenetic analyses

We conducted phylogenetic analyses using collected sequences of each gene family (excluding pseudogenes). Protein sequences were initially aligned using ClustalW 1.83 with the default options and MEGA Version 5.0 [47,48] for manual alignment corrections. The amino acid alignments were then used to guide the alignments of nucleotide coding sequences (CDSs). Phylogenetic trees were constructed based on the bootstrap neighbor-joining (NJ) method with a Kimura 2-parameter model by MEGA 5.0. The stability of internal nodes was assessed using bootstrap analysis with 1000 replicates (Figure 2 and see Additional file 1: Figure S1).

### Synteny analyses

Syntenic information for chromosome segments that include genes in the isoflavonoid biosynthesis pathway within the soybean genome was collected from the Plant Genome Duplication Database [49]. To establish synteny between soybeans and *M. truncatula*, target genes and flanking region genes extending 100 kb in each direction were blasted against*M. truncatula* genome sequences, and alignments with an E-value < 1e^−10^ were considered significant matches.

### Calculating K_S_ and dating the duplication event

Protein sequences of the gene pairs were aligned using Jalview, and the results were used to guide CDS alignments in Pal2Nal [50]. K_S_, the number of synonymous substitutions per site, was calculated using the CodeML program in PAML 4.3 with all alignment gaps excluded [36].

In dating segmental duplication events, six or fewer consecutive anchor points on each side of the isoflavonoid synthesis genes were chosen and used to calculate the average K_S_ after the minimum and maximum were removed. The approximate date of the segmental duplication event was calculated using the mean synonymous substitution rate (λ), which was 6.1 × 1e^−9^ for Fabaceae according to the formula T = Ks/2λ [51].

### Analysis of polymorphism and positive selection

Several identified gene copies within each gene family participating in isoflavonoid biosynthesis were chosen and sequenced in 33 Chinese soybean accessions, the collection locations of which are described in Cheng et al. [52]. Seeds from all of the accessions were obtained from Germplasm Storage of the Chinese National Center for Soybean Improvement (Nanjing Agricultural University, Nanjing, China). The sequenced regions of these genes were the most polymorphic regions based on 31 resequencing wild and cultivated soybean genomes [53]. For multi-copy genes, PCR primers were designed for specific regions flanking the sequencing regions, and sequencing primers were designed for sequencing regions (see Additional file 3: Table S3). PCR was conducted in a 50-μL reaction volume using KOD FX Neo polymerase (Toyobo, Japan) for 1 cycle of 3 min at 94°C; followed by 33 cycles of 30 s at 94°C for denaturation, 30 s at the annealing temperature for the respective primer pairs and 1 min at 68°C for extension; followed by 1 cycle of 10 min at 68°C using a PTC-225 thermal cycler (MJ Research, Watertown, MA). The PCR products were purified using the AxyPrep DNA Gel Extraction Kit (AxyGEN, Hangzhou China) and then sequenced on an ABI 3100A automated sequencer. All DNA sequences have been submitted to the GenBank databases (accession numbers KJ010826-KJ010858 and KM012193-KM012842).

The average pairwise nucleotide sequence diversity parameter (π) was calculated with DnaSP v5.0 using the Jukes and Cantor correction [54]. To determine whether any of these enzymes exhibited evidence of positive selection, we calculated the ratio of nonsynonymous (d_N_) to synonymous (d_S_) nucleotide substitution rates (d_N_/d_S_). We also used a maximum likelihood (ML) method to reveal the sites with significant positive selection. The ω ratio was calculated using the CodeML procedure in the phylogenetic analysis from PAML [36]. The likelihood ratio test (LRT) statistic for positive selection was conducted based on the comparison of M7 and M8 codon substitution models.

## Abbreviations

PAL: Phenylalanine ammonia-lyase; CHR: Chalcone reductase; IFS: Isoflavone synthase; WGD: Whole genome duplication; mya: Million years ago; 4CL: 4-coumarate: CoA ligase; CHS: Chalcone synthase; CHI: Chalcone isomerase; IOMT: Isoflavone O-methyltransferase; IFR: Isoflavone reductase; C4H: Cinnamate 4-hydroxylase; MRCA: Most recent common ancestor.

## Competing interests

The authors declare that they have no competing interests.

## Authors’ contributions

Conceived and designed the experiments: QY DY SC. Performed the experiments: SC JW HC. Analyzed the data: SC JW. Contributed reagents/materials/analysis tools: SC JW HC DY. Wrote the paper: QY SC JW. All authors read and approved the final manuscript.

## Supplementary Material

Additional file 1: Figure S1 The phylogenetic trees of genes in the isoflavonoid synthesis pathway from 7 species. Each species was labeled with different shapes as shown in the figure. Genes reported in NCBI are highlighted with a blue triangle. The genes sequenced in our study are highlighted with a red dot. The red circles at the nodes represent ancestral genes in the MRCA of legumes. The red triangles and rectangles represent ancestral genes in the MRCA of dicots and those of dicots and monocots, respectively. The nodes with bootstrapping lower than 50% are not shown. Each red square bracket represents one recent segmental duplication. a, *C4H* gene family. b, *4CL* gene family. c, *CHS* gene family. d, *CHI* gene family. e, *CHR* gene family. f, *IFS* gene family. g, *IOMT* gene family. h, *IFR* gene family. Click here for file

Additional file 2: Figure S2 Synteny analysis for genes in the isoflavonoid synthesis pathway. Green solid lines represent chromosomes of soybean. Target genes are indicated by red arrows. a, *C4H* gene family. b, *4CL* gene family. c, *CHS* gene family. d, *CHR* gene family. e, *CHI* gene family. f, *IFS* gene family. g, *IOMT* gene family. h, *IFR* gene family. Click here for file

Additional file 3: Table S1 All species used and their online databases. **Table S2.** The sequences used to conduct BLAST. **Table S3.** Primers used in this study. Click here for file

## References

[B1] Wang XQ Structure, function, and engineering of enzymes in isoflavonoid biosynthesis Funct Integr Genomic 2011 11 1 13 22 10.1007/s10142-010-0197-9 21052759

[B2] Dixon RA Harrison MJ Paiva NL The isoflavonoid phytoalexin pathway - from enzymes to genes to transcription factors Physiol Plantarum 1995 93 2 385 392

[B3] Winkel-Shirley B Flavonoid biosynthesis. A colorful model for genetics, biochemistry, cell biology, and biotechnology Plant Physiol 2001 126 2 485 493 11402179 10.1104/pp.126.2.485 PMC1540115

[B4] Mudge J Cannon SB Kalo P Oldroyd GE Roe BA Town CD Young ND Highly syntenic regions in the genomes of soybean, Medicago truncatula, and Arabidopsis thaliana BMC Plant Biol 2005 5 14 16102170 10.1186/1471-2229-5-15 PMC1201151

[B5] Yan HH Mudge J Kim DJ Shoemaker RC Cook DR Young ND Comparative physical mapping reveals features of microsynteny between Glycine max, Medicago truncatula, and Arabidopsis thaliana Genome 2004 47 1 141 155 15060611 10.1139/g03-106

[B6] Lee JM Bush AL Specht JE Shoemaker RC Mapping of duplicate genes in soybean Genome 1999 42 5 829 836

[B7] Pfeil BE Schlueter JA Shoemaker RC Doyle JJ Placing paleopolyploidy in relation to taxon divergence: a phylogenetic analysis in legumes using 39 gene families Syst Biol 2005 54 3 441 454 16012110 10.1080/10635150590945359

[B8] Fawcett JA Maere S Van de Peer Y Plants with double genomes might have had a better chance to survive the Cretaceous-Tertiary extinction event Proc Natl Acad Sci U S A 2009 106 14 5737 5742 19325131 10.1073/pnas.0900906106 PMC2667025

[B9] Shoemaker RC Schlueter J Doyle JJ Paleopolyploidy and gene duplication in soybean and other legumes Curr Opin Plant Biol 2006 9 2 104 109 16458041 10.1016/j.pbi.2006.01.007

[B10] Doyle JJ Egan AN Dating the origins of polyploidy events New Phytol 2010 186 1 73 85 20028472 10.1111/j.1469-8137.2009.03118.x

[B11] Schlueter JA Dixon P Granger C Grant D Clark L Doyle JJ Shoemaker RC Mining EST databases to resolve evolutionary events in major crop species Genome 2004 47 5 868 876 15499401 10.1139/g04-047

[B12] Schmutz J Cannon SB Schlueter J Ma JX Mitros T Nelson W Hyten DL Song QJ Thelen JJ Cheng JL Xu D Hellsten U May GD Yu YS Sakurai T Umezawa T Bhattacharyya MK Sandhu D Valliyodan B Lindquist E Peto M Grant D Shu SQ Goodstein D Barry K Futrell-Griggs M Abernathy B Du JC Tian ZX Zhu LC Genome sequence of the palaeopolyploid soybean Nature 2010 463 7278 178 183 20075913 10.1038/nature08670

[B13] Shoemaker RC Polzin K Labate J Specht J Brummer EC Olson T Young N Concibido V Wilcox J Tamulonis JP Kochert G Boerma HR Genome duplication in soybean (Glycine subgenus soja) Genetics 1996 144 1 329 338 8878696 10.1093/genetics/144.1.329 PMC1207505

[B14] Gill N Findley S Walling JG Hans C Ma JX Doyle J Stacey G Jackson SA Molecular and chromosomal evidence for allopolyploidy in soybean Plant Physiol 2009 151 3 1167 1174 19605552 10.1104/pp.109.137935 PMC2773056

[B15] Vitkup D Kharchenko P Wagner A Influence of metabolic network structure and function on enzyme evolution Genome Biol 2006 7 5 R39 16684370 10.1186/gb-2006-7-5-r39 PMC1779518

[B16] Cork JM Purugganan MD The evolution of molecular genetic pathways and networks Bioessays 2004 26 5 479 484 15112228 10.1002/bies.20026

[B17] Wright KM Rausher MD The evolution of control and distribution of adaptive mutations in a metabolic pathway Genetics 2010 184 2 483 U261 19966064 10.1534/genetics.109.110411 PMC2828727

[B18] Ramsay H Rieseberg LH Ritland K The correlation of evolutionary rate with pathway position in plant terpenoid biosynthesis Mol Biol Evol 2009 26 5 1045 1053 19188263 10.1093/molbev/msp021

[B19] Rausher MD Miller RE Tiffin P Patterns of evolutionary rate variation among genes of the anthocyanin biosynthetic pathway Mol Biol Evol 1999 16 2 266 274 10028292 10.1093/oxfordjournals.molbev.a026108

[B20] Lu YQ Rausher MD Evolutionary rate variation in anthocyanin pathway genes Mol Biol Evol 2003 20 11 1844 1853 12885963 10.1093/molbev/msg197

[B21] Rausher MD Lu YQ Meyer K Variation in constraint versus positive selection as an explanation for evolutionary rate variation among anthocyanin genes J Mol Evol 2008 67 2 137 144 18654810 10.1007/s00239-008-9105-5

[B22] Clotault J Peltier D Soufflet-Freslon V Briard M Geoffriau E Differential selection on carotenoid biosynthesis genes as a function of gene position in the metabolic pathway: a study on the carrot and dicots PLoS One 2012 7 6 e38724 22737218 10.1371/journal.pone.0038724 PMC3377682

[B23] Ramos-Onsins SE Puerma E Balana-Alcaide D Salguero D Aguade M Multilocus analysis of variation using a large empirical data set: phenylpropanoid pathway genes in Arabidopsis thaliana Mol Ecol 2008 17 5 1211 1223 18221273 10.1111/j.1365-294X.2007.03633.x

[B24] Yang YH Zhang FM Ge S Evolutionary rate patterns of the Gibberellin pathway genes BMC Evol Biol 2009 9 206 19689796 10.1186/1471-2148-9-206 PMC2794029

[B25] Yu G Olsen KM Schaal BA Molecular evolution of the endosperm starch synthesis pathway genes in rice (Oryza sativa L.) and its wild ancestor, O. rufipogon L Mol Biol Evol 2011 28 1 659 671 20829346 10.1093/molbev/msq243

[B26] Rausher MD The evolution of genes in branched metabolic pathways Evolution 2013 67 1 34 48 23289560 10.1111/j.1558-5646.2012.01771.x

[B27] Flowers JM Sezgin E Kumagai S Duvernell DD Matzkin LM Schmidt PS Eanes WF Adaptive evolution of metabolic pathways in Drosophila Mol Biol Evol 2007 24 6 1347 1354 17379620 10.1093/molbev/msm057

[B28] Whitt SR Wilson LM Tenaillon MI Gaut BS Buckler ES Genetic diversity and selection in the maize starch pathway Proc Natl Acad Sci U S A 2002 99 20 12959 12962 12244216 10.1073/pnas.202476999 PMC130568

[B29] Moore RC Purugganan MD The evolutionary dynamics of plant duplicate genes Curr Opin Plant Biol 2005 8 2 122 128 15752990 10.1016/j.pbi.2004.12.001

[B30] Cusack BP Wolfe KH When gene marriages don’t work out: divorce by subfunctionalization Trends Genet 2007 23 6 270 272 17418444 10.1016/j.tig.2007.03.010

[B31] Blanc G Wolfe KH Functional divergence of duplicated genes formed by polyploidy during Arabidopsis evolution Plant Cell 2004 16 7 1679 1691 15208398 10.1105/tpc.021410 PMC514153

[B32] Lindermayr C Mollers B Fliegmann J Uhlmann A Lottspeich F Meimberg H Ebel J Divergent members of a soybean (Glycine max L.) 4-coumarate : coenzyme A ligase gene family - primary structures, catalytic properties, and differential expression Eur J Biochem 2002 269 4 1304 1315 11856365 10.1046/j.1432-1033.2002.02775.x

[B33] Ralston L Subramanian S Matsuno M Yu O Partial reconstruction of flavonoid and isoflavonoid biosynthesis in yeast using soybean type I and type II chalcone isomerases Plant Physiol 2005 137 4 1375 1388 15778463 10.1104/pp.104.054502 PMC1088328

[B34] Lavin M Herendeen PS Wojciechowski MF Evolutionary rates analysis of Leguminosae implicates a rapid diversification of lineages during the tertiary Syst Biol 2005 54 4 575 594 16085576 10.1080/10635150590947131

[B35] Cheng H Wang J Chu SS Yan HL Yu DY Diversifying selection on flavanone 3-hydroxylase and isoflavone synthase genes in cultivated soybean and its wild progenitors PLoS One 2013 8 1 e54154 23342093 10.1371/journal.pone.0054154 PMC3546919

[B36] Yang ZH PAML 4: phylogenetic analysis by maximum likelihood Mol Biol Evol 2007 24 8 1586 1591 17483113 10.1093/molbev/msm088

[B37] Deavours BE Liu CJ Naoumkina MA Tang YH Farag MA Sumner LW Noel JP Dixon RA Functional analysis of members of the isoflavone and isoflavanone O-methyltransferase enzyme families from the model legume Medicago truncatula Plant Mol Biol 2006 62 4–5 715 733 17001495 10.1007/s11103-006-9050-x PMC2862459

[B38] Olson-Manning CF Lee CR Rausher MD Mitchell-Olds T Evolution of flux control in the glucosinolate pathway in Arabidopsis thaliana Mol Biol Evol 2013 30 1 14 23 22923463 10.1093/molbev/mss204 PMC3525143

[B39] Dhaubhadel S Gijzen M Moy P Farhangkhoee M Transcriptome analysis reveals a critical role of CHS7 and CHS8 genes for isoflavonoid synthesis in soybean seeds Plant Physiol 2007 143 1 326 338 17098860 10.1104/pp.106.086306 PMC1761968

[B40] Dall’Olio GM Laayouni H Luisi P Sikora M Montanucci L Bertranpetit J Distribution of events of positive selection and population differentiation in a metabolic pathway: the case of asparagine N-glycosylation BMC Evol Biol 2012 12 98 22731960 10.1186/1471-2148-12-98 PMC3426484

[B41] Yu O McGonigle B Metabolic engineering of isoflavone biosynthesis Adv Agron 2005 86 147 190

[B42] Jung W Yu O Lau SMC O’Keefe DP Odell J Fader G McGonigle B Identification and expression of isoflavone synthase, the key enzyme for biosynthesis of isoflavones in legumes (vol 18, pg 211, 2000) Nat Biotechnol 2000 18 5 559 559 10.1038/72671 10657130

[B43] Zhang JM Dean AM Brunet F Long MY Evolving protein functional diversity in new genes of Drosophila Proc Natl Acad Sci U S A 2004 101 46 16246 16250 15534206 10.1073/pnas.0407066101 PMC528974

[B44] Civetta A Positive selection within sperm-egg adhesion domains of fertilin: An ADAM gene with a potential role in fertilization Mol Biol Evol 2003 20 1 21 29 12519902 10.1093/molbev/msg002

[B45] Johnson ME Viggiano L Bailey JA Abdul-Rauf M Goodwin G Rocchi M Eichler EE Positive selection of a gene family during the emergence of humans and African apes Nature 2001 413 6855 514 519 11586358 10.1038/35097067

[B46] Kondrashov FA Rogozin IB Wolf YI Koonin EV Selection in the evolution of gene duplications Genome Biol 2002 3 2 RESEARCH0008 11864370 10.1186/gb-2002-3-2-research0008 PMC65685

[B47] Tamura K Peterson D Peterson N Stecher G Nei M Kumar S MEGA5: molecular evolutionary genetics analysis using maximum likelihood, evolutionary distance, and maximum parsimony methods Mol Biol Evol 2011 28 10 2731 2739 21546353 10.1093/molbev/msr121 PMC3203626

[B48] Thompson JD Higgins DG Gibson TJ Clustal-W - improving the sensitivity of progressive multiple sequence alignment through sequence weighting, position-specific gap penalties and weight matrix choice Nucleic Acids Res 1994 22 22 4673 4680 7984417 10.1093/nar/22.22.4673 PMC308517

[B49] Tang HB Bowers JE Wang XY Ming R Alam M Paterson AH Perspective - Synteny and collinearity in plant genomes Science 2008 320 5875 486 488 18436778 10.1126/science.1153917

[B50] Suyama M Torrents D Bork P PAL2NAL: robust conversion of protein sequence alignments into the corresponding codon alignments Nucleic Acids Res 2006 34 W609 W612 16845082 10.1093/nar/gkl315 PMC1538804

[B51] Lynch M Conery JS The evolutionary fate and consequences of duplicate genes Science 2000 290 5494 1151 1155 11073452 10.1126/science.290.5494.1151

[B52] Cheng H Yu O Yu DY Polymorphisms of IFS1 and IFS2 gene are associated with isoflavone concentrations in soybean seeds Plant Sci 2008 175 4 505 512

[B53] Lam HM Xu X Liu X Chen WB Yang GH Wong FL Li MW He WM Qin N Wang B Li J Jian M Wang JA Shao GH Wang J Sun SSM Zhang GY Resequencing of 31 wild and cultivated soybean genomes identifies patterns of genetic diversity and selection Nat Genet 2010 42 12 1053 U1041 21076406 10.1038/ng.715

[B54] Librado P Rozas J DnaSP v5: a software for comprehensive analysis of DNA polymorphism data Bioinformatics 2009 25 11 1451 1452 19346325 10.1093/bioinformatics/btp187

