# Supplementary figures and images for: Evolutionary study of the isoflavonoid pathway based on multiple copies analysis in soybean
Source: BMC Genet. 2014 Jun 24;15:76. doi: 10.1186/1471-2156-15-76 (PMC4076065; doi:10.1186/1471-2156-15-76)

Fig. S1

**a C4H**

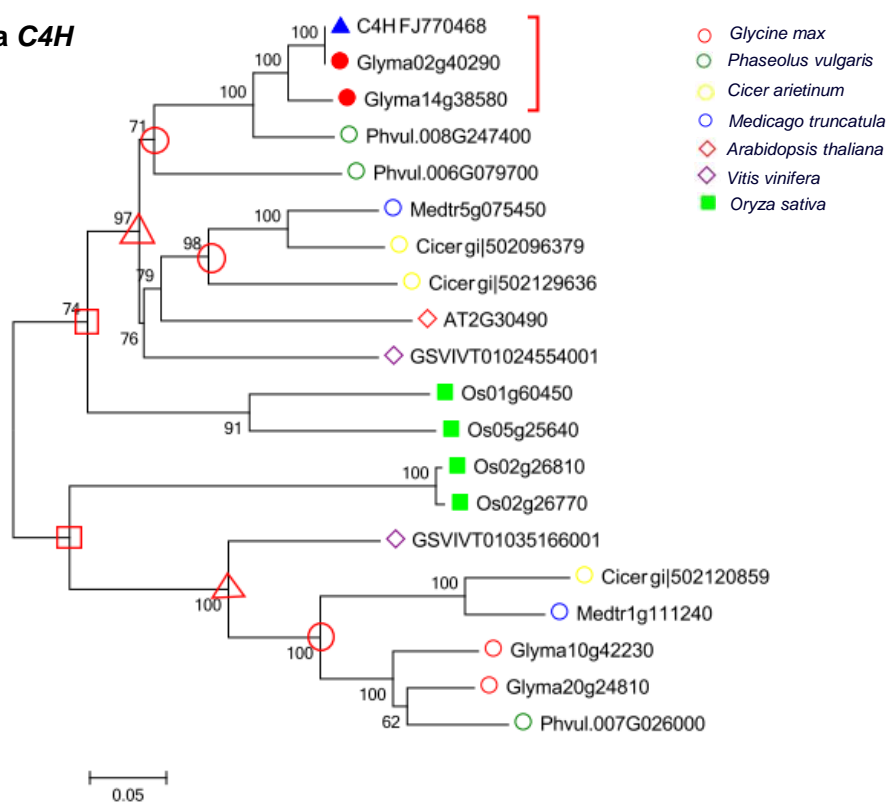

**b 4CL**

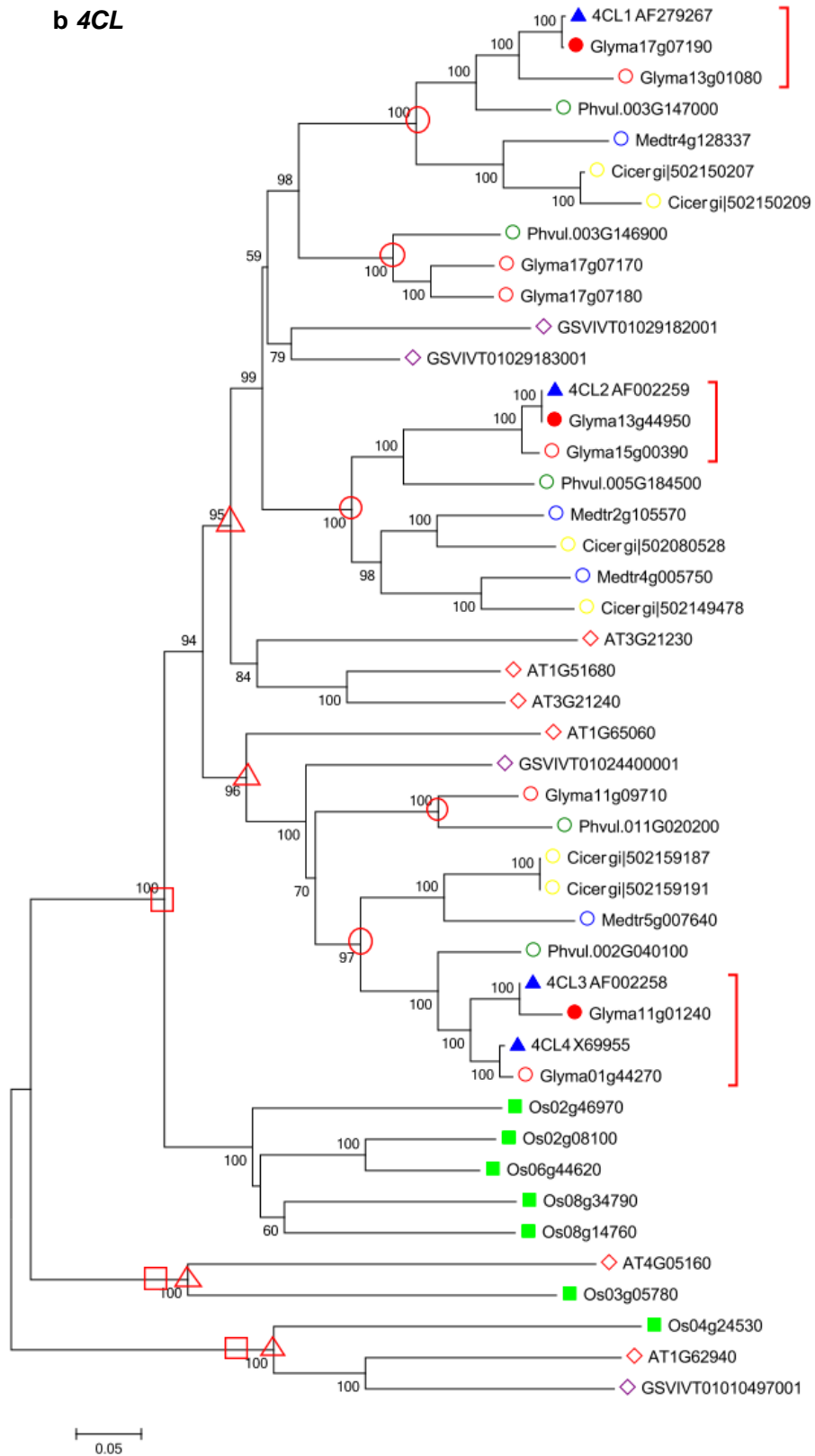

c CHS

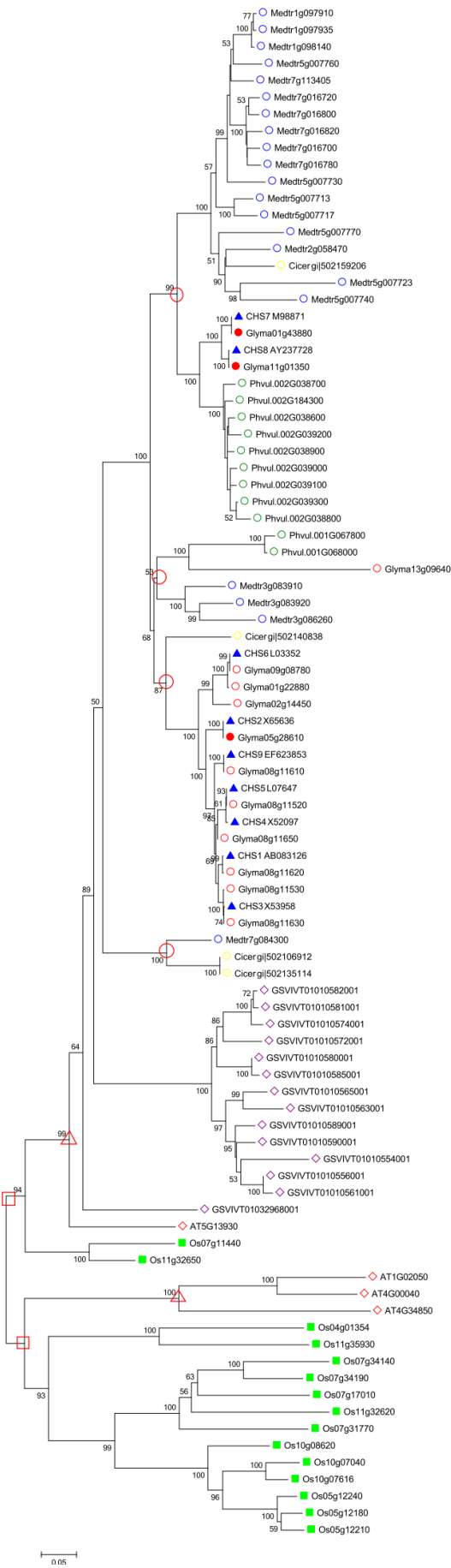

**d CHI**

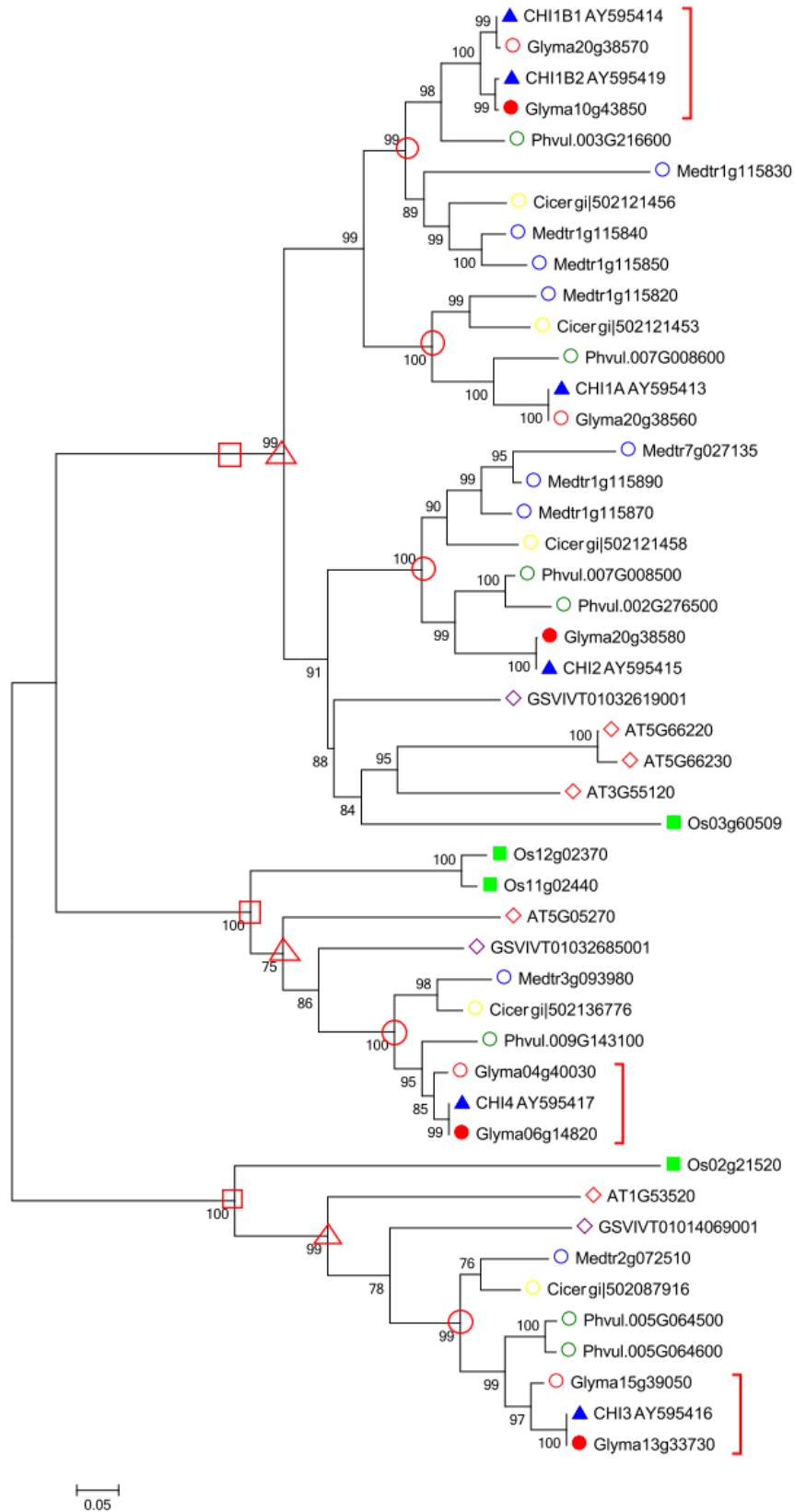

**e CHR**

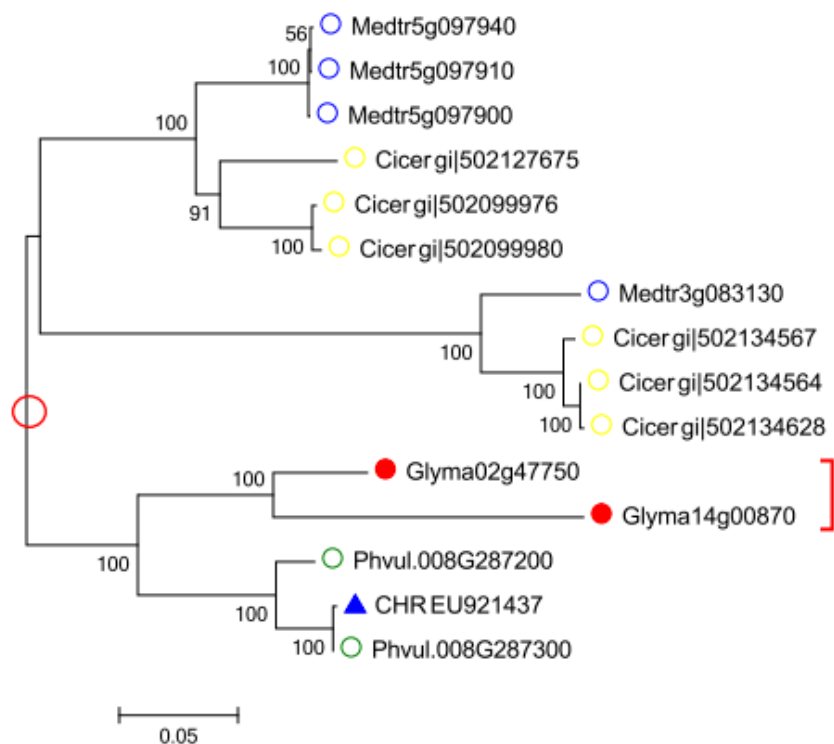

**f IFS**

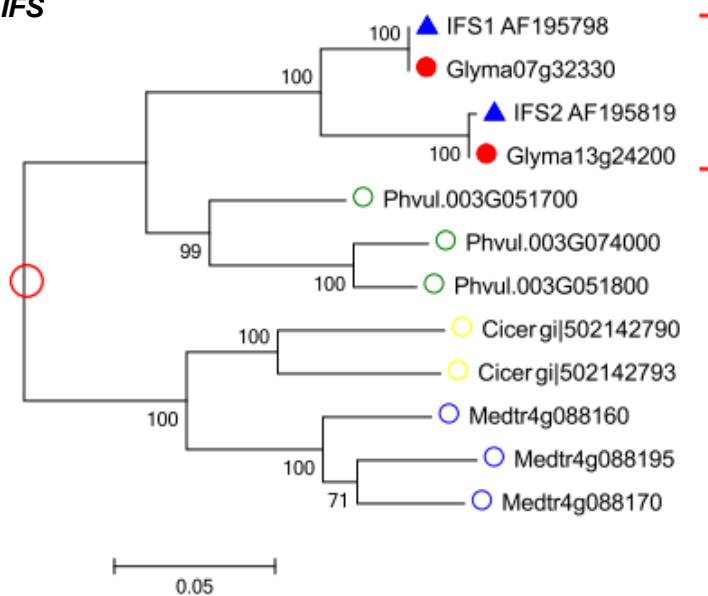

**g IOMT**

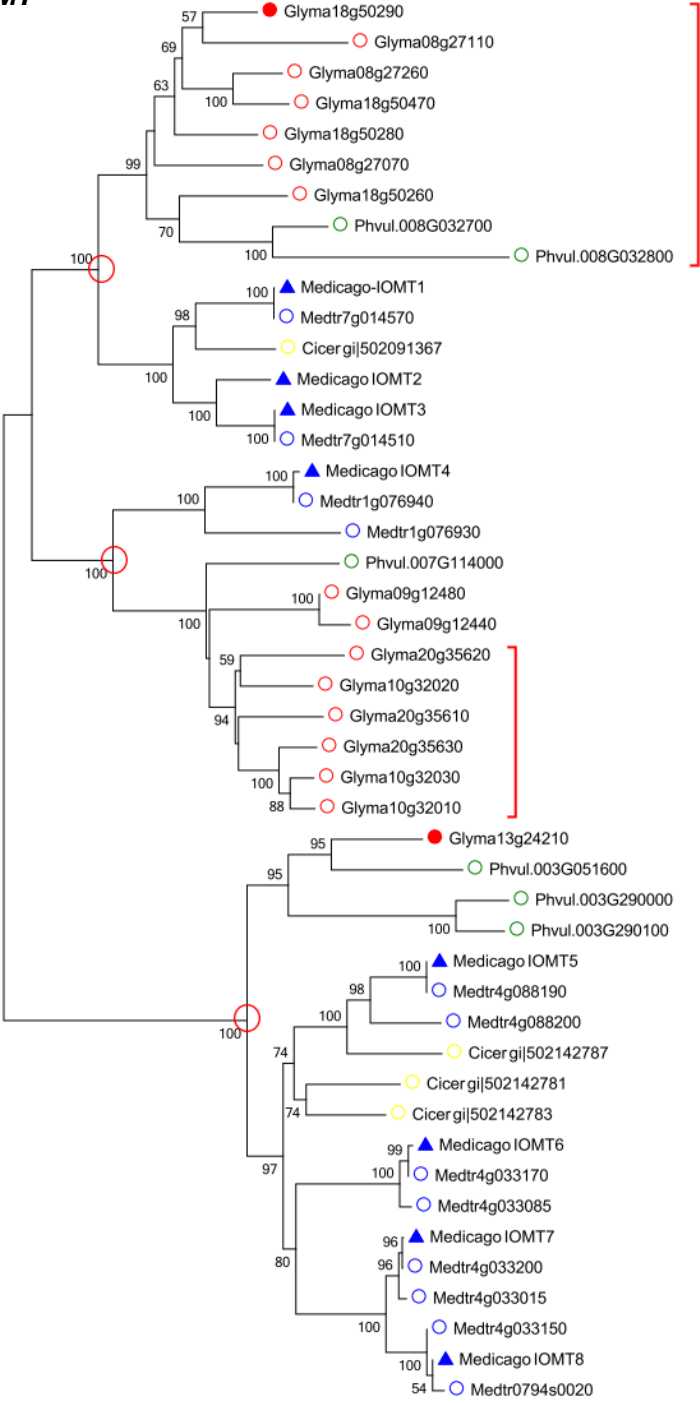

0.05

h *IFR*

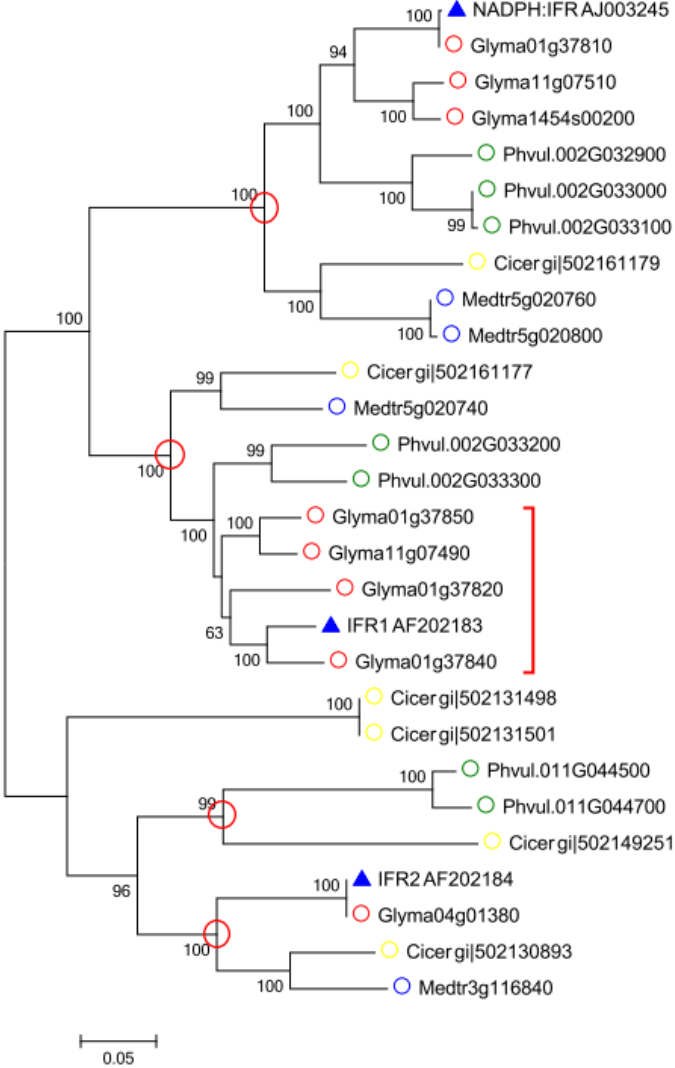

Supplement: Additional file 1: Figure S1 — The phylogenetic trees of genes in the isoflavonoid synthesis pathway from 7 species. Each species was labeled with different shapes as shown in the figure. Genes reported in NCBI are highlighted with a blue triangle. The genes sequenced in our study are highlighted with a red dot. The red circles at the nodes represent ancestral genes in the MRCA of legumes. The red triangles and rectangles represent ancestral genes in the MRCA of dicots and those of dicots and monocots, respectively. The nodes with bootstrapping lower than 50% are not shown. Each red square bracket represents one recent segmental duplication. a, C4H gene family. b, 4CL gene family. c, CHS gene family. d, CHI gene family. e, CHR gene family. f, IFS gene family. g, IOMT gene family. h, IFR gene family. [file 1471-2156-15-76-S1.pdf]
