# Supplementary material for: Evolutionary study of the isoflavonoid pathway based on multiple copies analysis in soybean
Source: BMC Genet. 2014 Jun 24;15:76. doi: 10.1186/1471-2156-15-76 (PMC4076065; doi:10.1186/1471-2156-15-76)
Supplement: Additional file 2: Figure S2 — Synteny analysis for genes in the isoflavonoid synthesis pathway. Green solid lines represent chromosomes of soybean. Target genes are indicated by red arrows. a, C4H gene family. b, 4CL gene family. c, CHS gene family. d, CHR gene family. e, CHI gene family. f, IFS gene family. g, IOMT gene family. h, IFR gene family. [file 1471-2156-15-76-S2.pdf]

Fig. S2

a ***C4H***

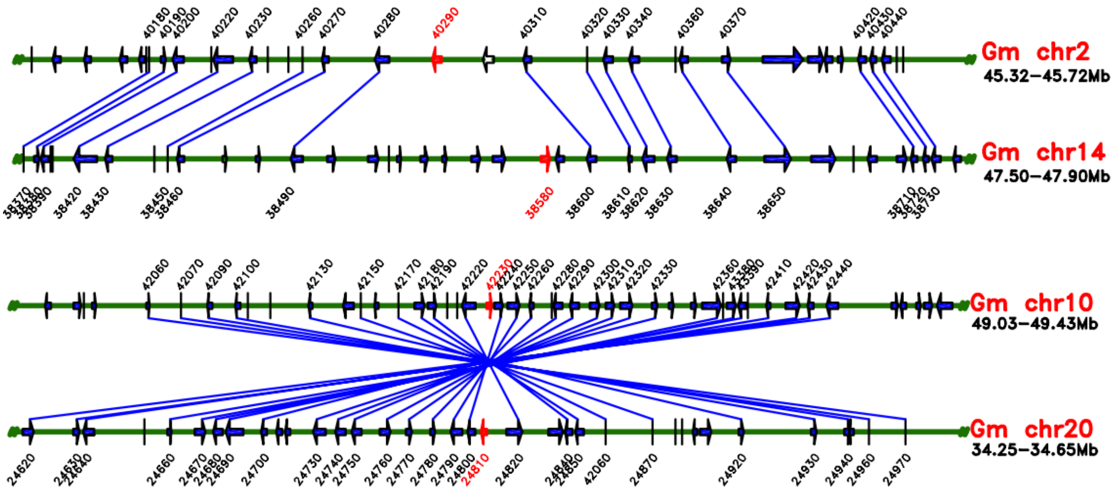

b ***4CL***

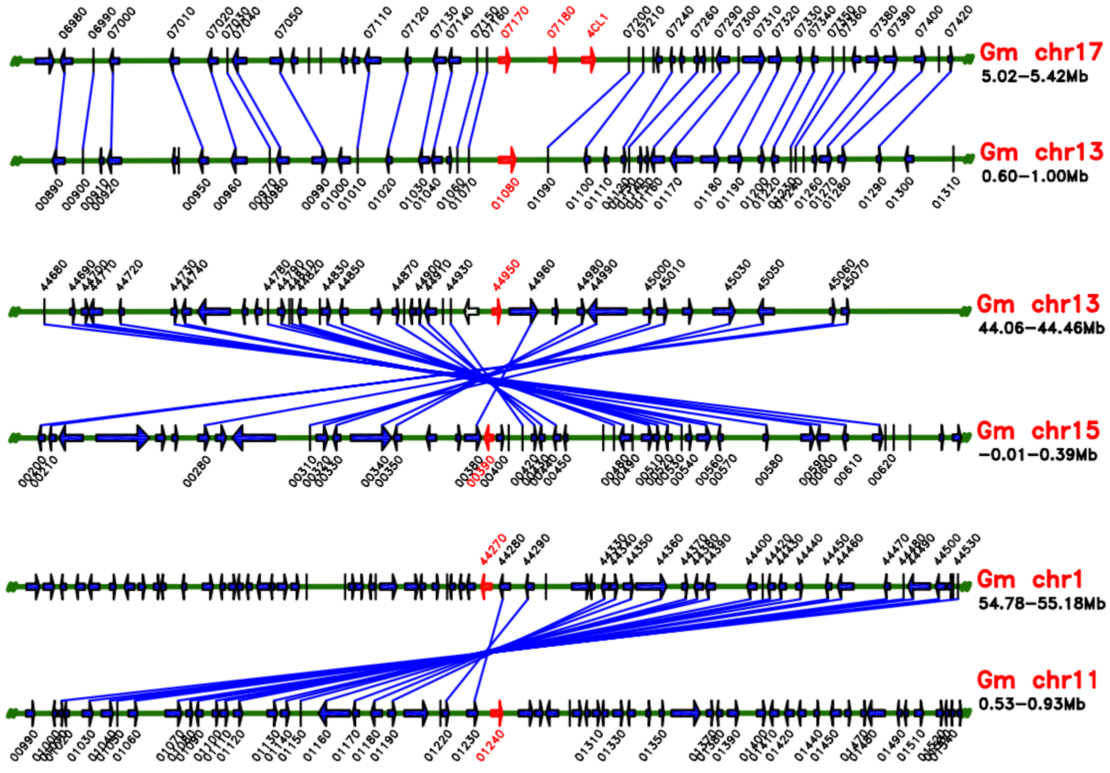

c *CHS*

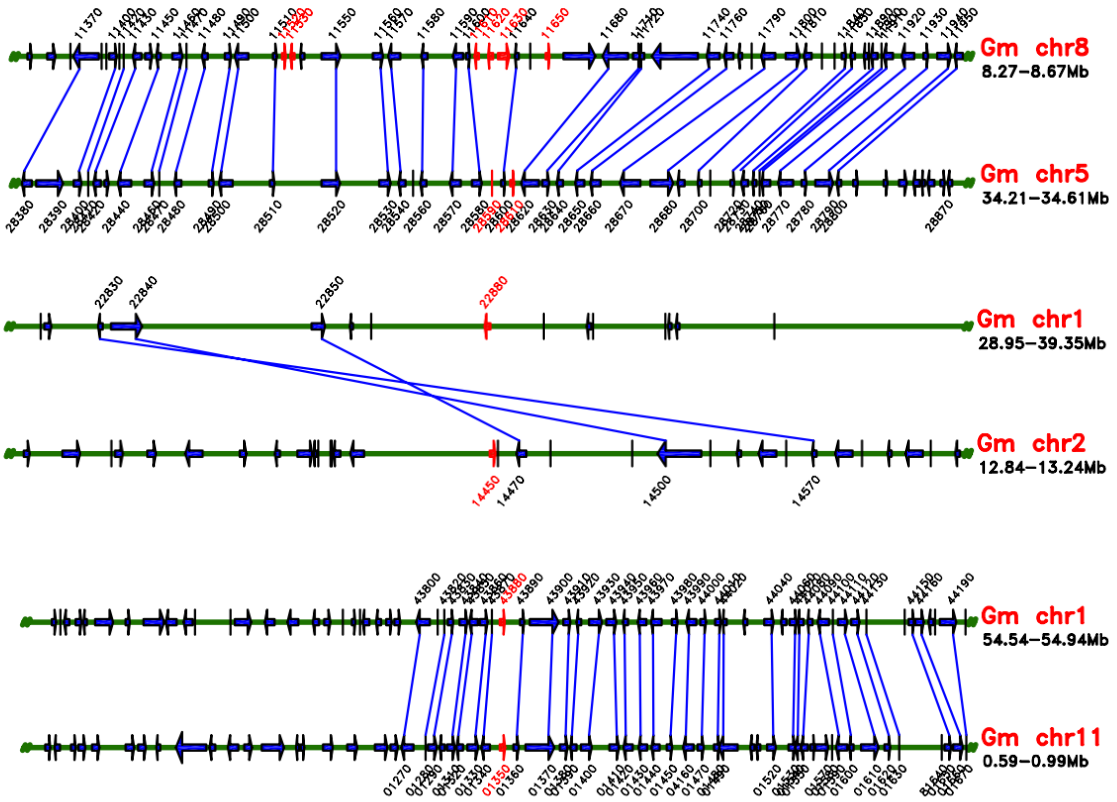
Gm chr8
8.27–8.67Mb
Gm chr5
34.21–34.61Mb
Gm chr1
28.95–39.35Mb
Gm chr2
12.84–13.24Mb
Gm chr1
54.54–54.94Mb
Gm chr11
0.59–0.99Mb

d *CHR*

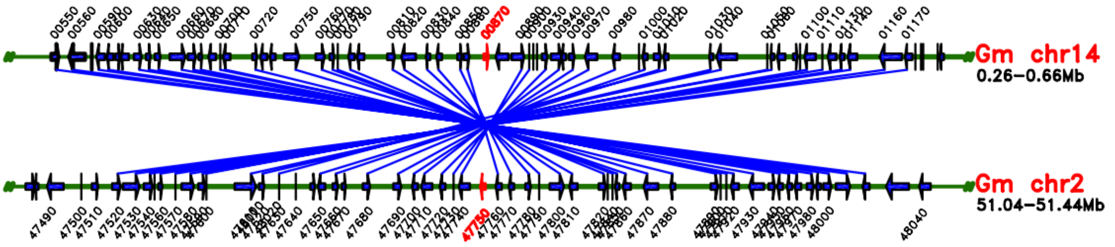
Gm chr14
0.26–0.66Mb
Gm chr2
51.04–51.44Mb

e *CHI*

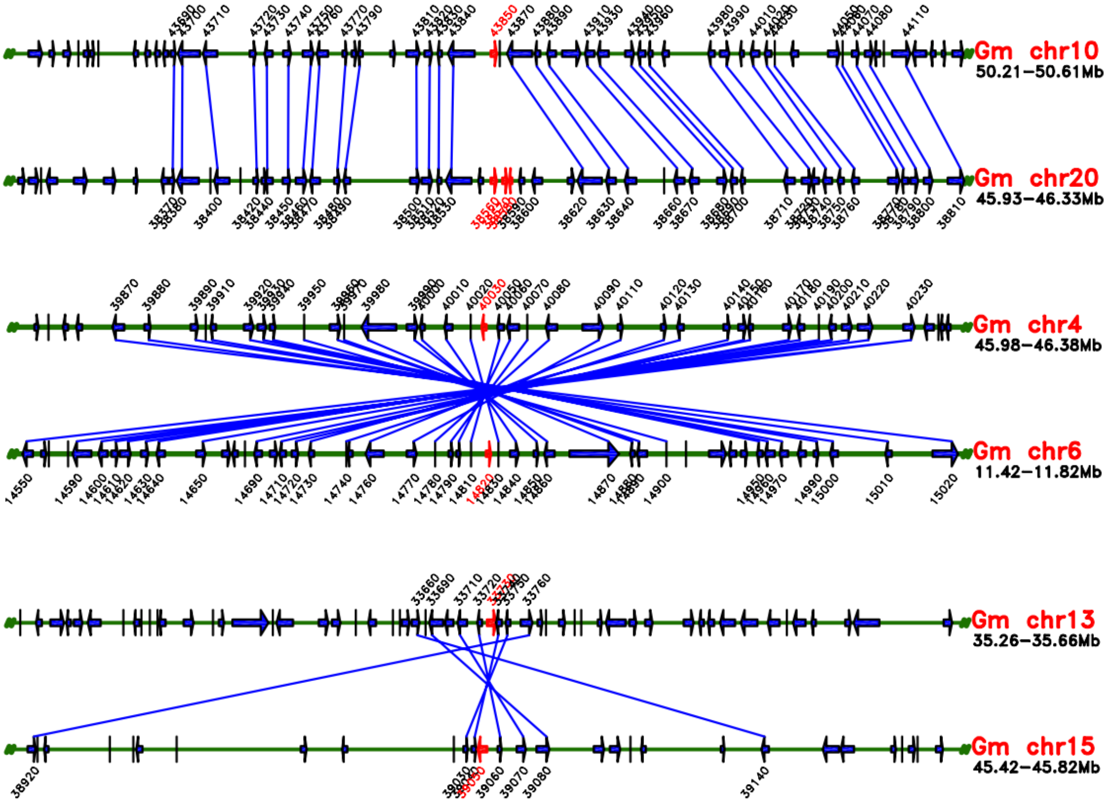
Gm chr10
50.21–50.61Mb
Gm chr20
45.93–46.33Mb
Gm chr4
45.98–46.38Mb
Gm chr6
11.42–11.82Mb
Gm chr13
35.26–35.66Mb
Gm chr15
45.42–45.82Mb

f *IFS*

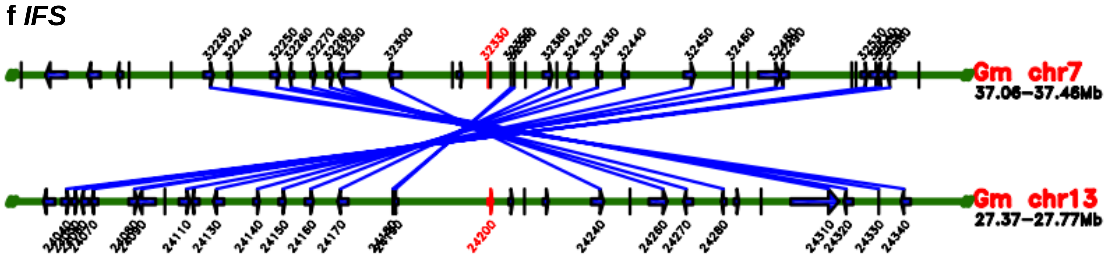
Gm chr7
37.06–37.46Mb
Gm chr13
27.37–27.77Mb

g *IOMT*

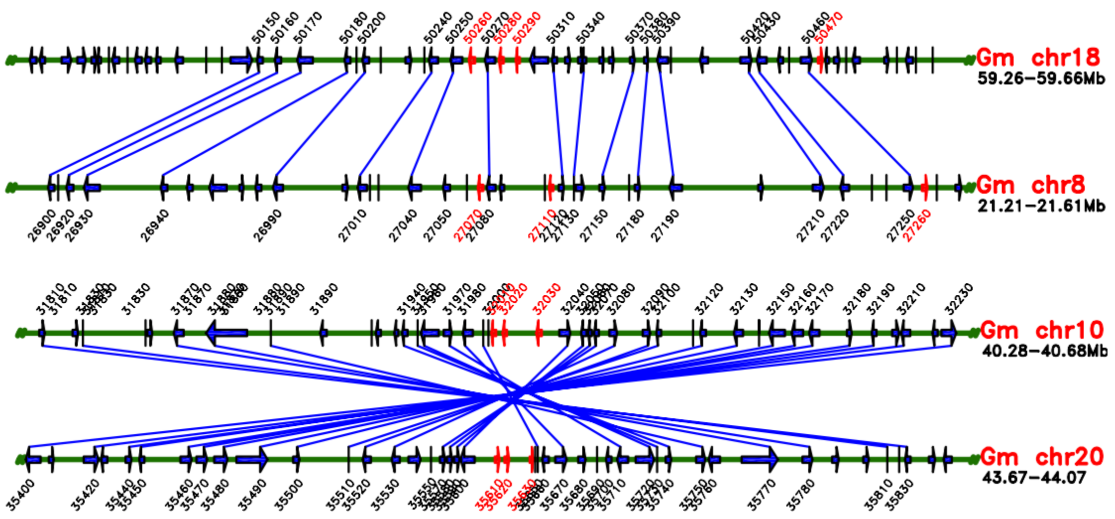
Gm chr18
59.26–59.66Mb
Gm chr8
21.21–21.61Mb
Gm chr10
40.28–40.68Mb
Gm chr20
43.67–44.07

h *IFR*

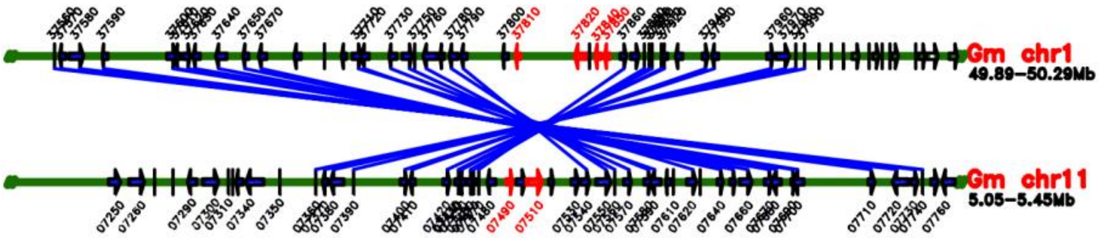
Gm chr1
49.89–50.29Mb
Gm chr11
5.05–5.45Mb
