# Supplementary material for: Evolutionary study of the isoflavonoid pathway based on multiple copies analysis in soybean
Source: BMC Genet. 2014 Jun 24;15:76. doi: 10.1186/1471-2156-15-76 (PMC4076065; doi:10.1186/1471-2156-15-76)
Supplement: Additional file 3: Table S1 — All species used and their online databases. Table S2. The sequences used to conduct BLAST. Table S3. Primers used in this study. [file 1471-2156-15-76-S3.pdf]

Supplementary tables

Table S1 All species used and their online databases

Species	Online databases
Glycine max	Phytozome v9.0 (ftp://ftp.jgi-psf.org/pub/compgen/phytozome/v9.0/Gmax/)
Phaseolus vulgaris	Phytozome v9.0 (ftp://ftp.jgi-psf.org/pub/compgen/phytozome/v9.0/Pvulgaris/)
Cicerarietinum	NCBI (ftp://ftp.ncbi.nlm.nih.gov/genomes/Cicer_arietinum/)
Medicagotruncatula	Mt4.0v1 (http://medicago.jcvi.org/medicago/display.php?pageName=General&section=Download)
Arabidopsis thaliana	TAIR10 (ftp://ftp.arabidopsis.org/Genes/TAIR10_genome_release/)
Vitisvinifera	Phytozome v9.0 (ftp://ftp.jgi-psf.org/pub/compgen/phytozome/v9.0/Vvinifera/)
Oryza sativa	Phytozome v9.0 (ftp://ftp.jgi-psf.org/pub/compgen/phytozome/v9.0/Osativa/)

Table S2 The sequences used to conduct BLAST

Gene	GenBank accession no.
PAL1	X52953
PAL2	GQ358921
C4H	FJ770468
4CL1	AF279267
4CL2	AF002259
4CL3	AF002258
4CL4	X69955
CHS1	AB083126
CHS2	X65636
CHS3	X53958
CHS4	X52097
CHS5	L07647
CHS6	L03352
CHS7	M98871
CHS8	AY237728
CHS9	EF623853
CHI1A	AY595413
CHI1B1	AY595414
CHI1B2	AY595419
CHI2	AY595415
CHI3	AY595416
CHI4	AY595417
CHR	EU921437
IFS1	EU391490
IFS2	EU391494
IOMT	AY942159
IFR1	AF202183
IFR2	AF202184
NADPH-IFR	AJ003245

Table S3 Primers used in this study

Enzyme	Gene locus	PCR primers (5'-3')	Sequencing primers (5'-3')
PAL	Glyma19g36620	F:5-TTAGTGATGACGGACCTTA	F-TGCAACATGCTTTCATCA
		R:5-CTCCTCCAAATGCCTC	
	Glyma03g33890	F:5'- GCGACATGCTTCGTTCA-	F- TCAAGGGAGCTGAAATT
		R:5'- TGACATTGAAGGAACATTTG	
	Glyma10g06600	F:5- CGTCCTACGATTTCCCG	R- TAGTCCACAAGCACTTGCC
		R: 5'-TGATCTTCCCTTGGCACA	
C4H	Glyma14g38580	F-CATGCCACCAGCCTTAC	F-CATGCCACCAGCCTTAC
		R-AATGGTGGAATGCTTGAG	
	Glyma02g40290	F- CTCTATGGTCGATTGAGTG	F- CTCTATGGTCGATTGAGTG
		R- GATCATGTGGTGGAGTCTA	
4CL	Glyma17g07190	F- CAAGCAAAGTCGCAAAT	F-TCTCCTCCTACACCAACAA
		R- CTCCTGTAAATGCTATCCA	
	Glyma13g44950	F- ATCTCCAGGCTATCTATCA	F-AAGAGGGCGTGGAACAGG
		R- AACGTGAGAACTTGTCATG	
	Glyma11g01240	F- AAGACAATGACAACGGTAGCTG	F-AAGACAATGACAACGGTAGCTG
		R- ACGGAAGCACGCATAGGA	
CHS	Glyma05g28610	F-GCTATCTAGGATGGTGAGTG	F-GGATCAGGTGATAAGTCAAT
		R-ATCAAAAGACAGAATAAGCC	
	Glyma01g43880	F-CGTCGATCTGCGAATCAT	F-TTTCAGGTGACAAGTCTATG
		R-ATTTCCTCCTCATCTCATCC	
	Glyma11g01350	F-CAGTCGTAGTAGACGGTGGA	F-ATGGCACCTTCTTTGGAT
		R-GCCATCCAGGGAGGTAA	
CHR	Glyma02g47750	F-TCTTTATCGCCAAATCTTAC	F-TCTTTATCGCCAAATCTTAC
		R-AGAGCACGAGGCACATT	
	Glyma14g00870	F- CTTCAACTAGAGTACTTGGACC	R- TAGAGTGGGAGCTGCATAG
		R- TAGAGTGGGAGCTGCATAG	
CHI	Glyma10g43850	F- CAAAACAGGTCGAGACAGA	F- CTTGTGGCTATAGGTGTGC
		R- GCATTGCTCAGGCTTTAT	
	Glyma20g38580	F- TTTCCGTCCGTAACCTCT	F- TTTCCGTCCGTAACCTCT
		R- ATGCATGAGTAAGACTAACCG	
	Glyma06g14820	F-CAACTTGCTCTACCTACCCACC	F-CACCGTTAGTGCATGTATTA
		R-AACACAAAACACAGACCCCAAG	
	Glyma13g33730	F- CTGAGATTAACTACGAGGCAC	F- TATTGGTGTCAAGGTCTATGC
		R- GGAGAATATAGGCTACGGC	
I0MT	Glyma13g24210	F- TGGCAACAGCACTGAAGAG	F- TGGCAACAGCACTGAAGAG
		R- CTGCATCGGCAGAGGG	
	Glyma18g50290	F- AATCAATGGCCGTAATGC	F- AATCAATGGCCGTAATGC
		R- GTCCATTGGACGATATTGTTT	
IFR	Glyma01g37840	F- ATCAAGGCCACAAACTC	R- GATGGTGTTTAGGAGCA
		R- GATGGTGTTTAGGAGCA	
	Glyma04g01380	F- GTAGGAGCCTGGTCTGGT	F- GTAGGAGCCTGGTCTGGT
		R- CGGTGCAACAGAGTATCACTA	
